# Chiral Plasmonic Printing of Discrete Hierarchical Chiral Au@Chiral Ag Semi‐Core‐Shell Nanorods With Chiral Plasmon‐Enhanced Photocatalysis

**DOI:** 10.1002/advs.76451

**Published:** 2026-07-20

**Authors:** Yaxin Cao, Haoyu Li, Mengli Wu, Shengshi Fan, Shenli Wang, Haibo Zhou, Guangchao Zheng

**Affiliations:** ^1^ Colloidal Physics Group Key Laboratory of Materials Physics School of Physics and Laboratory of Zhongyuan Light Ministry of Education Zhengzhou University Zhengzhou P. R. China; ^2^ College of Food Science and Technology Henan University of Technology Zhengzhou P. R. China; ^3^ Institute of Pharmaceutical Analysis College of Pharmacy Jinan University Guangzhou P. R. China; ^4^ Institute of Quantum Materials and Physics Henan Academy of Sciences Zhengzhou P. R. China

**Keywords:** chiral hot electrons, chiral plasmonic coupling, hierarchical chirality, nanorods, photocatalysis

## Abstract

Understanding and harnessing the interaction between circularly polarized light (CPL) and nanocrystal morphology is crucial for designing advanced functional materials, yet a chemically chiral ligand‐free pathway to fabricate structurally defined hybrid chiral nanostructures has been elusive. Herein, we report a novel chiral plasmonic printing strategy along with its underlying theoretical model, which employs the spin angular momentum of CPL as a chemically chiral‐ligand‐free stimulus to drive the asymmetric deposition of silver onto discrete chiral Au nanorods. This approach enables the programmable synthesis of hierarchically chiral Au@chiral Ag semi‐core‐shell nanostructures, whose chiroptical properties are synergistically governed by the intrinsic chirality of the Au core and the handedness of the incident CPL, and can be precisely modulated by illumination time. Theoretical modeling and experimental characterization jointly demonstrate that the chiral Ag shell dictates the circularly polarized photocatalytic activity performance via chiral hot‐electron generation, as verified in the model reduction of 4‐nitrophenol. This work not only establishes a versatile solution‐phase route for fabricating complex chiral metallic nanostructures but also provides a predictive “light‐handedness‐to‐nanostructure‐to‐function” framework, offering new opportunities for the rational design of advanced materials in asymmetric catalysis and chiral photonics.

## Graphical abstract

Chiral plasmonic printing uses CPL as a chiral stimulus to guide asymmetric Ag deposition on chiral Au NRs seeds. Under CPL, hot carriers reduce Ag^+^, and the process is controlled by both the CPL handedness and the chiral SPR. L‐dc Au generates more hot electrons under LCP than RCP, while D‐dc Au shows the opposite, revealing chiral photon‐selective interactions that affect the catalytic rate.

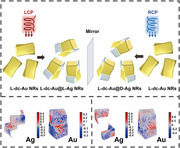

## 1 Introduction

Chirality enables matter to interact with CPL, allowing photon energy to be used at the molecular scale [1, 2, 3]. Natural chiral molecules, however, show weak responses in the UV region. Plasmonic nanomaterials overcome this by concentrating light into nanoscale hotspots via localized surface plasmon resonance (SPR), acting as optical reactors that amplify signals and enable applications, creating the field of chiral plasmonics [3, 4, 5]. Fabrication of chiral nanomaterials follows two paths, including precise but costly top‐down lithography and scalable wet‐chemistry methods using chemically chiral ligands [6, 7]. Wet‐chemistry methods can produce nanostructures with strong, stable chiroptical responses [8]. However, the use of chemically chiral ligands has given rise to a dichotomy between two perspectives. One is advocating chirality guided by the coupling of chiral molecules and SPR, and another is emphasizing chirality guided by helical plasmonic nanostructures [9, 10, 11, 12, 13, 14].

In this context, chiral plasmonic printing has emerged as a disruptive paradigm, leveraging the spin angular momentum (SAM) of CPL as a controllable and non‐chemical chiral stimulus via chiral SPR [15, 16]. Each photon of CPL carries a quantized SAM of ±ħ, dictating its left‐ or right‐handedness [17]. This intrinsic property can be directly imparted to plasmonic nanomaterials during light‐matter interactions, driving asymmetric photochemical processes such as site‐selective metal deposition or directional nanoparticle self‐assembly [18, 19, 20, 21]. This method establishes a chiral molecule‐free route for the preparation of chiral plasmonic nanostructures. The handedness of these structures is determined solely by the SAM of the incident CPL, thereby eliminating the dependency on chiral templates. The mechanistic foundation of this technology is the efficient coupling between a photon's SAM and plasmonic hot carriers [22]. For instance, under CPL illumination, the hot‐electron distribution on achiral plasmonic seeds acquires an intrinsically chiral geometry. This asymmetric energy landscape directs the asymmetry‐selective reduction of metal precursors on achiral seeds, yielding nanostructures with strong chiroptical response. Consequently, the handedness of the resulting 3D nanohelicoids and other complex architectures is dictated by the SAM of the incident CPL [23]. These works confirm the feasibility of photon‐to‐matter chirality transfer, establishing a new paradigm for the optical control of chiral morphology.

Chiral plasmon‐enhanced photocatalysis merges chiroptics with plasmonics to improve both the selectivity and efficiency of catalytic processes, opening a promising avenue [24, 25, 26]. Chiral plasmonic nanostructures, such as Au helicoids, Au@CeO_2_ helical nanorods, and twisted Pd nanoarrays on chiral Au nanorods, exhibit strong chiroptical responses and SPR, enabling asymmetric hot‐carrier generation under CPL [27, 28, 29]. These nanostructures have shown excellent performance in reactions such as N_2_ photofixation, CO_2_ reduction, and H_2_ evolution, with photocatalytic efficiency significantly amplified when the handedness of CPL matches the chirality of the nanostructure [26, 27, 30, 31, 32]. Advantages of this approach include tunable chiroptical responses, circularly polarized photocatalytic activity, and compatibility with visible to near‐infrared light. Despite recent advances in chiral photocatalysts, hierarchical chiral heterostructured nanocatalysts, featuring a chiral core combined with a chiral shell, remain scarce.

In this study, we employed a chiral plasmonic printing strategy to fabricate discrete hierarchical chiral Au@chiral Ag core‐shell nanorods (NRs) using discrete chiral Au NRs (dc‐Au NRs) as seeds. Serving as chiral templates, these dc‐Au NRs (L‐dc‐Au and D‐dc‐Au NRs) guided the asymmetric deposition of an Ag shell under CPL illumination, yielding semi‐core‐shell structures with hierarchical chirality. Unlike previously reported work that relies entirely on achiral gold nanoparticles (NPs) [18], in this study, we employ discrete chiral Au nanorods (L‐dc‐Au and D‐dc‐Au NRs) as seeds. These chiral seeds possess an intrinsic circular dichroism (CD) signal and a chiral plasmonic near‐field distribution, providing a new “chiral stimulus” for subsequent CPL‐directed silver deposition. Moreover, while previous chiral nanostructures consist of an achiral core and a chiral Ag shell, we design analogous chiral metal architectures featuring a chiral Au core and a chiral Ag shell, thereby achieving hierarchical chirality. Under 405 nm CPL (either left‐ or right‐handed) excitation and in the presence of citrate as a reducing agent, Ag^+^ ions were reduced to metallic Ag by hot carriers generated via the chiral localized SPR of the dc‐Au NRs. The Ag deposition process was synergistically controlled by the handedness of the incident CPL and the plasmonic near‐field distribution of the dc‐Au NRs, ultimately resulting in discrete chiral Au@ chiral Ag core‐shell NRs with well‐defined hierarchical chirality. The resulting nanostructures exhibited circular‐polarization‐dependent and circularly polarized photocatalytic activity in the model reduction of 4‐nitrophenol. Theoretical simulations of the hot‐electron generation rate (ΔHE Rate) further revealed that the chiral Ag shell dominates the helicity‐dependent hot‐carrier profile, while the chiral Au core modulates the selective interaction with CPL, collectively regulating the photocatalytic efficiency. This work offers a controllable new strategy for the low‐cost, efficient preparation of complex chiral metallic nanostructures in solution, holding considerable potential for applications in chiral catalysis, photonics, and the design of advanced functional materials.

## 2 Results and Discussion

**FIGURE 1:**
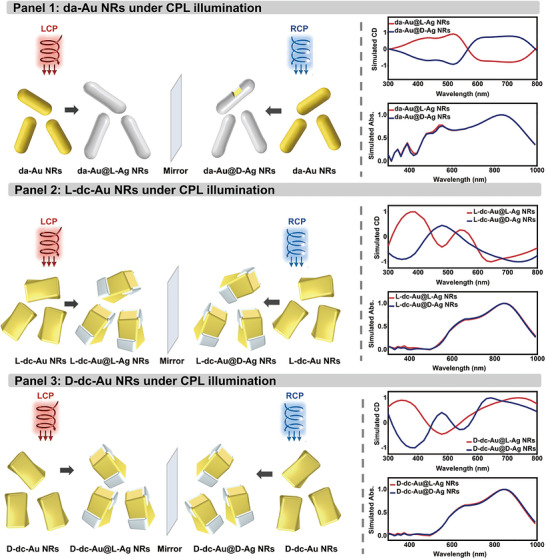
Schematic of the photochemical Ag growth on the surfaces of da‐Au NRs (Panel 1), L‐dc‐Au NRs (Panel 2), and D‐dc‐Au NRs (Panel 3) solution under CPL illumination, along with their corresponding simulated CD and abs. spectra.

Discrete achiral Au NRs (da‐Au NRs) were synthesized following our established protocol, and their transmission electron microscopy (TEM) images are shown in Figure S1 [14, 33]. A one‐step ligand exchange process was first performed to replace hexadecyltrimethylammonium chloride (CTAC) on the da‐Au NRs with poly (sodium 4‐styrenesulfonate) (PSS). Sodium citrate and AgNO_3_ were then introduced into the dc‐Au NR dispersion, followed by illumination with 405 nm CPL (LCP or RCP) generated from an LED source using polarization optics (Power density: 2.83 mW cm^−2^) (**Panel 1** in Figure 1). Under CPL excitation, the chiral SPR of da‐Au NRs drives collective electron oscillations and produces hot carriers, which promote citrate oxidation and Ag^+^ reduction. The resulting silver atoms are deposited asymmetrically onto the da‐Au NRs surface, guided by the handedness of chiral SPR, leading to the formation of da‐Au@ chiral Ag core‐shell NRs (da‐Au@c‐Ag NRs) (Figure S2b,c). The extinction spectra of da‐Au@c‐Ag NRs showed characteristic longitudinal and transverse SPR peaks at 447 and 764 nm, respectively (Figure S2e,g); their chiroptical activity was confirmed by CD spectroscopy (Figure S2d,f), which is confirmed by the simulated CD spectra and UV spectra (**Panel 1** in Figure 1). This process demonstrates the introduction of chiral SPR and subsequent asymmetric deposition on initially achiral nanoparticles via CPL excitation [18, 34]. A natural question then arises: *how would hot‐electron generation and directional growth behave when CPL interacts with intrinsically chiral nanostructures*?

**FIGURE 2:**
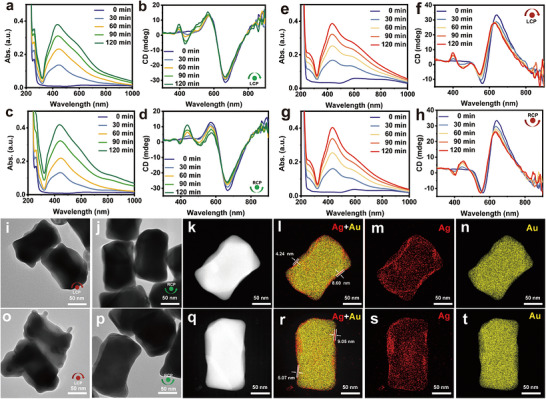
Absorbance and CD spectra of L‐dc‐Au@c‐Ag and D‐dc‐Au@c‐Ag NRs dispersions after 405 nm CPL illumination for 120 min. (a, b) L‐dc‐Au@c‐Ag NRs under LCP; (c, d) L‐dc‐Au@c‐Ag NRs under RCP; (e, f) D‐dc‐Au@c‐Ag NRs under LCP; (g, h) D‐dc‐Au@c‐Ag NRs under RCP. TEM and STEM characterization of L‐dc‐Au@c‐Ag and D‐dc‐Au@c‐Ag NRs synthesized under 405 nm CPL for 120 min: (i, j) TEM images of L‐dc‐Au@c‐Ag NRs. TEM images of L‐dc‐Au@c‐Ag NRs obtained after LCP (i) and RCP (j). (k) L‐dc‐Au@D‐Ag NRs: HAADF‐STEM (RCP, 120 min). (l–n) EDS elemental maps showing the distribution of (l) L‐dc‐Au@D‐Ag NRs, (m) Ag, and (n) Au, respectively. (o, p) TEM images of D‐dc‐Au@c‐Ag NRs. TEM images of D‐dc‐Au@c‐Ag NRs obtained after LCP (o) and RCP (p). (q) D‐dc‐Au@L‐Ag NRs: HAADF‐STEM (LCP, 120 min). (r–t) EDS elemental maps showing the distribution of (r) D‐dc‐Au@L‐Ag NRs, (b) Ag, and (c) Au, respectively.

Following the above questions, we have conducted an experiment to irradiate dc‐Au NRs with CPL (**Panels 2** and **3** in Figure 1). Time‐resolved absorbance and CD spectra were collected to monitor CPL‐directed Ag deposition on dc‐Au NRs (Figure 2a–h). L‐ and D‐dc‐Au NRs were initially synthesized, and their well‐defined rod‐like morphology was confirmed by TEM (Figure S3a–d). Extinction spectra of the dc‐Au NRs featured characteristic longitudinal and transverse SPR peaks at approximately 594 and 784 nm, while chiroptical activity was confirmed by CD measurements (Figure S3e,f). The extinction spectra of L‐dc‐Au NRs under LCP illumination (L‐dc‐Au@L‐Ag NRs) are presented in Figure 2a. At the initial stage (0 min), no discernible CD signal was observed in the 200‐400 nm region, whereas two apparent CD signals were present at 594 and 784 nm. Subsequently, a distinct Cotton effect emerged and intensified, characterized by a positive peak near 394 nm, a negative peak at 438 nm, and a broad feature around 590 nm. The CD peaks at 394, 438, and 590 nm appeared, conforming to the formation of a chiral Ag shell on the surface of dc‐Au NRs (L‐dc‐Au@L‐Ag NRs) (Figure 2b). The chiroptical performance remained almost constant after heating the reaction to 40°C or 60°C for 2 h in the absence of light illumination (Figure S4), ruling out any significant photothermal contribution. This spectral pattern originates from coupled chiral SPR between a chiral gold core and a chiral silver shell, reflecting the dynamic morphological evolution of the silver shell. Control experiments with exogenous injection of L‐ or D‐cysteine (10^−5^ M) in the synthetic procedures showed no clear CD peak of the chiral Ag shell, revealing that trace amounts of residual chiral molecules on the surface of dc‐Au NRs seeds neither contribute to the chiroptical response nor limits the formation of the chiral Ag shell (see Figure S5). Along with the blue shift observed in the absorption spectrum, these changes in chiroptical activities are attributed to the inhomogeneous nucleation and growth of Ag.

Subsequently, we conducted the aforementioned experiment using RCP with the same laser wavelength range of 405 nm. Interestingly, under RCP excitation, the extinction peak of L‐dc‐Au NRs also exhibited a blue‐shift in wavelength, consistent with the behavior observed under LCP illumination (Figure 2c). Correspondingly, the CD spectrum of L‐dc‐Au NRs irradiated with RCP (L‐dc‐Au@D‐Ag NRs, Figure 2d) and L‐dc‐Au@L‐Ag NRs displayed mirror‐symmetric profiles across the 300–500 nm range. These spectral features include a mirror band at 394, 438, and 590 nm, attributed to the growth of an asymmetric silver shell. Meanwhile, the CD spectra ranging from 500 to 900 nm retain the spectral information of L‐dc‐Au NRs but experience a blue shift. These observed changes in both the CD and extinction spectra are further supported by theoretical simulations (**Panel 2** in Figure 1).

Under continuous CPL illumination, silver atoms were asymmetrically deposited onto the surfaces of L‐dc‐Au NRs in a manner dictated by the handedness of the incident light. CPL enantioselectively excites chiral hot electrons at chiral surface regions, which in turn steer the asymmetric growth of a semi‐chiral Ag shell, significantly modulating the chiroptical response of the resulting nanostructures. To address whether the process is truly plasmon‐enhanced, we performed control experiments on D‐dc‐Au NRs at 633 nm (near the longitudinal SPR of dc‐Au NRs at ∼594 nm). At 633 nm, we observed extensive self‐nucleation of Ag nanoparticles rather than seeded growth, attributed to highly efficient hot‐electron generation under resonant excitation that promotes rapid Ag^+^ reduction in solution (Figure S6). In contrast, at 405 nm, moderate hot‐electron flux favors kinetically controlled, site‐selective deposition on chiral seeds. Under 405 nm, Au undergoes interband transitions, which generate few hot electrons [35]. Thus, hot electrons from Au plasmons contribute little to chiral growth. Instead, hot electrons excited by the plasmon resonance of Ag likely drive the chiral growth of c‐Au NRs@c‐Ag [18]. In order to further confirm the plasmon‐mediated photocatalysis, we conducted direct photo‐excitation of the citrate‐Ag^+^ complex without Au NRs. As a result, the extinction peaks at 400 nm, and no significant CD signal confirms that photochemical reduction happened in this process. However, the chiroptical response of c‐Au NRs@c‐Ag originates from plasmon‐mediated photocatalysis rather than direct photoexcitation of the citrate‐Ag^+^ complex. Therefore, the process is plasmon‐mediated, not a simple non‐resonant photochemical pathway (Figure S7). The contrasting outcomes at 405 nm (controlled chiral shell growth) vs. 633 nm (self‐nucleation) further confirm that the process is plasmon‐mediated but optimized under off‐resonant conditions, where the hot‐electron flux is sufficiently moderate to favor heterogeneous seeded growth over homogeneous nucleation. This is consistent with recent reports showing that chirally distributed hot electrons generated by CPL can drive site‐selective metal deposition even at wavelengths away from the LSPR peak, highlighting the importance of kinetic control in plasmon‐mediated chiral growth [23, 36]. We also performed a control experiment using Au NRs encapsulated in a thick insulating SiO_2_ shell (Figure S8). Under CPL illumination, these Au@SiO_2_ NRs exhibited an Ag absorption peak at ∼400 nm but no detectable CD signal, revealing that hot‐electron transfer is essential for handedness‐selective deposition. The longitudinal and transverse SPR peaks of the L‐dc Au@c‐Ag nanorods both exhibit a distinct blue shift along with spectral broadening. This behavior is attributed to the significantly lower imaginary part of the dielectric function of the Ag shell compared to that of Au, which greatly reduces the imaginary component of the effective permittivity of the composite structure. The characteristic CD signals arise from the synergistic interplay between the intrinsic chirality of the L‐dc‐Au NRs and the deposited chiral Ag shell. The enantioselective deposition and reduction of silver ions on the dc‐Au NR surfaces are driven by LCP and RCP via chiral‐SPR‐generated hot carriers. This process effectively transduces the SAM of the CPL into the optical activity of the dc‐Au@c‐Ag nanostructures. Because the hot‐electron distribution under RCP excitation is mirror‐symmetric to that under LCP, silver atoms preferentially nucleate and grow on the corresponding opposite sides of the dc‐Au NRs, ultimately yielding an enantiomorphic “chiral Au NR semi‐core‐chiral Ag shell” hierarchical architecture (Figure 2j). This configuration induces pronounced plasmonic coupling between the chiral gold core and the semi‐chiral silver shell, further amplifying the chiroptical and catalytic properties of the hybrid nanostructure.

Subsequently, D‐dc‐Au NRs were irradiated with 405 nm LCP, which was also in situ monitored by the time‐resolution UV‐vis‐NIR and CD spectrum. The corresponding CD spectra exhibited the characteristic peaks at 394 and 438 nm, displaying a spectral line shape across 300‐500 nm that is nearly identical to that observed for L‐dc‐Au NRs under the same LCP excitation (Figure 2e,f). Moreover, both the negative and positive CD features around 600 nm underwent a blue shift, analogous to the coating of chiral silver shell on the dc‐Au NRs, a shift attributable to the intrinsic characteristic of the imaginary part of silver's dielectric constant relative to that of gold. These spectral variations in both the CD and extinction spectra are consistent with theoretical simulations and experimental observations (**Panel 3** in Figure 1). These results indicate that an identical chiroptical response of the deposited chiral Ag shell is obtained under identical RCP illumination, regardless of the intrinsic handedness (L or D) of the dc‐Au NRs. Under RCP illumination, the silver coating on D‐dc‐Au NRs follows a similar chiral electromagnetic field‐directed reduction and growth mechanism (Figure 2g,h). Notably, the chirality of the resulting Ag shell on D‐dc‐Au NRs under RCP matches that formed on L‐dc‐Au NRs under the same RCP illumination, while being mirror‐symmetric to the Ag shell grown on the same D‐dc‐Au NRs under LCP. We adjusted the laser power in the preliminary experiments, and the CD and absorbance spectra of c‐Au@Ag NRs dispersions after 0.226 mW cm^−2^ 405 nm RCP illumination for 60 min (Figure S9) and 0.453 mW cm^−2^ 405 nm RCP illumination for 120 min (Figure S10) are presented herein, where the spectra for D‐dc‐Au@Ag NRs and L‐dc‐Au@Ag NRs under the former illumination condition are shown in Figure S24 and those under the latter condition in Figure S25, respectively, with no significant changes observed under either of these two illumination conditions. Further experiments established the threshold power density for effective Ag shell growth at ∼2.83 mW/cm^2^. Below this value (e.g., 1.41 mW/cm^2^), the photon flux is insufficient to drive appreciable Ag reduction, leading to only weak evolution of the UV‐vis‐NIR absorbance and CD signals (Figure S11). Above the threshold, moderate increases in illumination intensity (e.g., 4.24 mW/cm^2^) yield growth kinetics and chiroptical properties nearly identical to those observed at the threshold (Figure S12). In addition, the g‐factor achieves a constant value after 120 min (Figure S13). Based on these control experiments, 2.83 mW cm^−2^ and 120 min were selected as the optimal deposition time under CPL.

TEM images further reveal asymmetric Ag deposition on the resulting nanostructures (Figure 2i,j,o,p). HAADF‐STEM and energy‐dispersive X‐ray spectroscopy (EDX) elemental mapping confirm an Au‐rich core accompanied by spatially inhomogeneous Ag distribution (Figure 2k–n,q–t), consistent with handedness‐dependent near‐field/hot‐carrier‐directed nucleation and growth, with Ag thickness varying from ∼4.2 to 8.6 nm in Figure 2l and from ∼5.1 to 9.1 nm in Figure 2r. EDX line‐scanning analysis further reveals an asymmetric thickness of the silver nanoshell (Figure S14). Theoretical simulations show that the semi‐core‐shell structure (Figure S15a,b) preserves the original chirality of the dc‐Au NRs, whereas the core‐shell structure (Figure S15c) leads to CD peaks reverse regardless of the Ag morphologies, further confirming the semi‐core‐shell configuration in this manuscript. Therefore, the chirality of c‐Au NRs@c‐Ag nanostructures originates from two cooperative factors, including the intrinsic chirality of the dc‐Au NR cores and the CPL‐induced asymmetric growth of the Ag shell. Control experiments without CPL in our previous work demonstrate that the Ag shell alone can inherit the core's chirality, producing symmetric CD signals at the longitudinal SPR position [33]. Under CPL illumination, the core provides a chiral template, and the CPL‐directed shell growth inherits the CD signals of the dc‐Au NRs while introducing additional programmable asymmetry. Thus, the intrinsic core chirality serves as the fundamental origin, and the CPL‐driven shell growth further tailors and enhances chiroptical activity.

**FIGURE 3:**
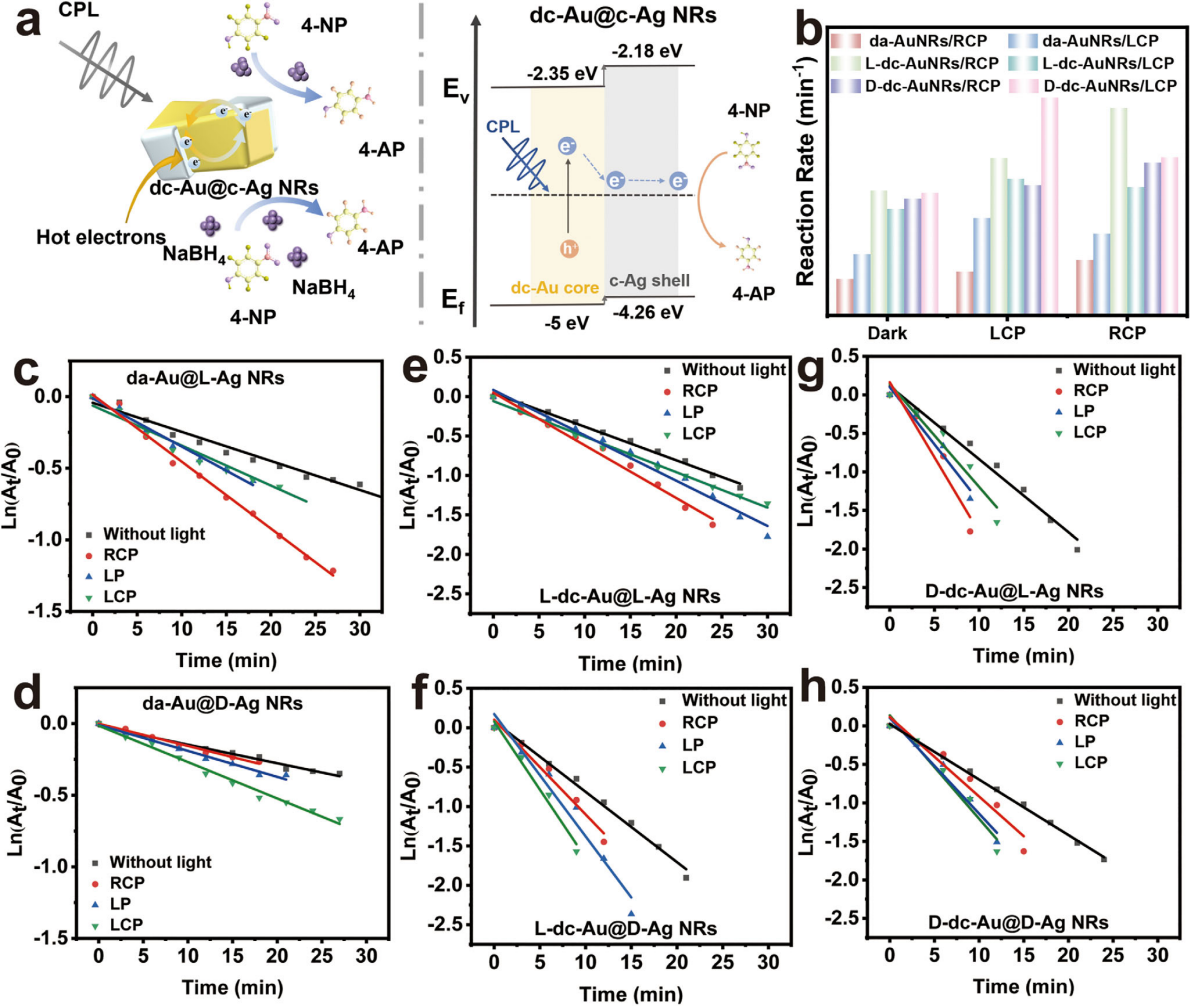
(a) Schematics of the Au@Ag nanostructure photocatalysts showing the nitro group reduction process. (b) Rate profiles of different materials under Dark, LCP, and RCP illumination. (c, d) Plot of Ln(A_t_/A_0_) as a function of time for the reaction catalyzed by da‐Au@L‐Ag NRs and da‐Au@D‐Ag NRs under different polarized light excitations, respectively. (e, f) Plot of Ln(A_t_/A_0_) as a function of time for the reaction catalyzed by L‐dc‐Au@L‐Ag NRs and L‐dc‐Au@D‐Ag NRs under different polarized light excitations, respectively. (g, h) Plot of Ln(A_t_/A_0_) as a function of time for the reaction catalyzed by D‐dc‐Au@L‐Ag NRs and D‐dc‐Au@D‐Ag NRs under different polarized light excitations, respectively.

To evaluate the chiroptical photocatalytic performance of the chiral plasmon‐printed chiral Au@ chiral Ag semi‐core‐shell nanostructures, the reduction of 4‐nitrophenol (4‐NP) to 4‐aminophenol (4‐AP) by excess NaBH_4_ was used as a model reaction (Figure 3a). The reaction progress was monitored by tracking the decay of the characteristic absorption band peak around 400 nm (Figures S17–S22), and the apparent rate constants (k) were obtained from pseudo‐first‐order kinetics, Ln(A_t_/A_0_) = −K_t_. Compared with the dark controls, illumination markedly accelerates the reaction for all catalysts, confirming a photoenhanced pathway. In contrast, da‐Au NRs and dc‐Au NRs exhibited negligible photocatalytic performance under identical conditions (Figure S16), confirming that the chiral Ag shell play a key role for the enhanced and polarized‐light‐dependent photocatalysis in such system.

Strikingly, The systematic analysis of the photocatalytic kinetics data reveals that the circularly polarized photocatalytic activity circular polarization‐dependent activity of these chiral Au@chiral Ag semi‐core‐shell nanostructures is predominantly governed by the handedness of the outer silver shell, with minimal influence from the intrinsic handedness of the gold nanorod core. Both L‐dc‐Au@L‐Ag and D‐dc‐Au@L‐Ag possess higher catalytic efficiency under RCP illumination in comparison to under LCP illumination (Figure 3e,g). Conversely, L‐dc‐Au@D‐Ag and D‐dc‐Au@D‐Ag perform more effectively in terms of catalytic efficiency under LCP illumination (Figure 3f,h). Similarly, da‐Au@L‐Ag has higher catalytic efficiency under RCP illumination than under LCP illumination (Figure 3c), while da‐Au@D‐Ag shows more effective catalytic efficiency under LCP illumination (Figure 3d). This pattern indicates that, regardless of whether the core is L‐ or D‐dc‐Au NRs or da‐Au NRs, the circularly polarized photocatalytic activity photocatalytic response to CPL is dictated solely by the handedness of the silver shell (Figure 3b). A comparative analysis indicates that the dc‐Au@c‐Ag NRs structure demonstrates significantly better catalytic performance than the da‐Au@c‐Ag NRs structure [37, 38]. Under ambient conditions, the spectra in Figure S23 show that while the absolute CD and absorption intensities decreased after one catalytic cycle (30 min, 1 recycle), the characteristic spectral features and derived g‐factor remained detectable, confirming that the chiroptical response of the dc‐Au@c‐Ag NRs is partially preserved after photocatalytic use.

This enhancement stems from the strong chiral plasmonic coupling between the intrinsically chiral Au core and the chiral Ag shell, which significantly enhances the local electromagnetic field. From a hot‐carrier perspective, such strong coupling redirects the generation and injection of energetic carriers, creating a helicity‐dependent hot‐carrier profile that differs from conventional circular dichroism [39, 40]. This mechanism further promotes the generation and injection efficiency of hot electrons. This trend indicates that the catalytic efficiency depends not only on the intrinsic chirality of the dc‐Au NRs core and chiral Ag shell, but also on the handedness of the polarized light illumination. In this model reaction, where light‐driven acceleration is commonly linked to SPR‐mediated pathways, the superior performance of dc‐Au core catalysts can be attributed to a synergistic combination of effects. On one hand, the dc‐Au NRs core provides a foundation of increased hot‐spot density; within this architecture, more efficient excitation of the coupled chiral Au‐Ag plasmon system further promotes chiral hot‐carrier generation and interfacial chiral hot electrons transfer during nitro‐group reduction. Concurrently, the chiral Ag shell itself supplies additional chiral hot electrons, thereby directly facilitating the charge‐transfer processes critical at the catalytic interface. This trend indicates that the catalytic efficiency depends not only on the intrinsic chirality of the dc‐Au NRs core and chiral Ag shell, but also on the handedness of the polarized light illumination.

**FIGURE 4:**
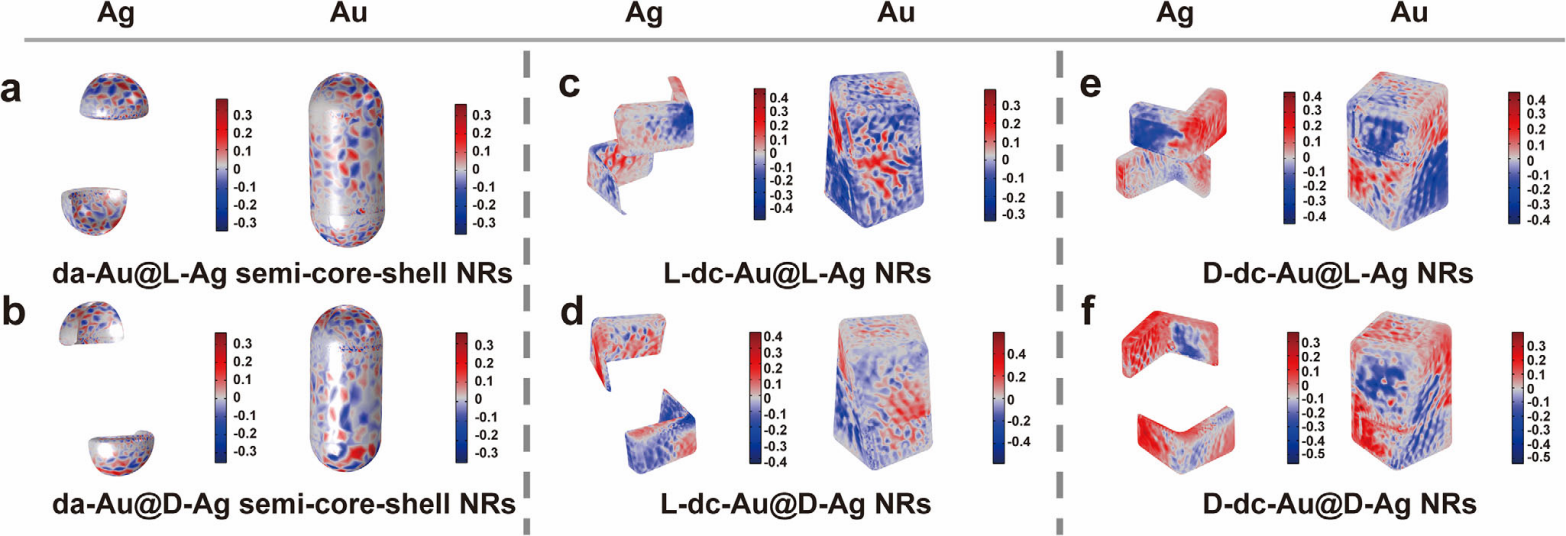
The differences in hot electron generation rates (HE Rate) under LCPL and RCPL for chiral nanoparticles: da‐Au@L‐Ag semi‐core‐shell NRs (a), da‐Au@D‐Ag semi‐core‐shell NRs (b), L‐dc‐Au@L‐Ag NRs (c), L‐dc‐Au@D‐Ag NRs (d), D‐dc‐Au@L‐Ag NRs (e), and D‐dc‐Au@D‐Ag NRs (f).

The extinction cross sections under LCP and RCP were calculated as defined in Note S1. The helicity‐dependent hot electron generation rate (HE Rate) (Figure S25) and electric field enhancement (Figure S26), the difference under LCP and RCP illumination (ΔHE Rate) of the chiral Au@Ag nanorod (NRs) structures, including da‐Au@L‐Ag semi‐core‐shell NRs, da‐Au@D‐Ag semi‐core‐shell NRs, L‐dc‐Au@L‐Ag NRs, L‐dc‐Au@D‐Ag NRs, D‐dc‐Au@L‐Ag NRs, D‐dc‐Au@D‐Ag NRs are systematically characterized (Figure 4) [36, 41]. We adopted a semi‐core‐shell for da‐Au@c‐Ag NRs to eliminate the morphological influence on the generation of hot electrons. HE rate distributions are calculated by the following formula:

(1) $$\begin{array}{c} HE_{\mathrm{rate}}\;\left(r\right)=\mathrm{lo}g_{10}\;\left(\frac{1}{4}\cdot \frac{2}{\pi^{2}}\cdot \frac{e^{2}E_{F}^{2}}{\hbar }\frac{\left(\hbar \omega -\Delta E_{\mathrm{bar}}\right)}{{\left(\hbar \omega \right)}^{4}}{\left|E_{\omega }\right|}^{2}\right) \end{array}$$

here, E_ω_ denotes the normal component of the electric field on the surface inside the nanoparticle, and E_F_ =   − 5 eV is the Fermi energy for gold, E_F_ =   − 4.26 eV is the Fermi energy for silver. Quantitative surface‐integrated ΔHE Rates for Ag and Au domains reveal distinct helicity‐dependent responses. The generation of HEs is largely influenced by the local electromagnetic field, thus, the surface distribution of ΔHE rate and ΔE is very similar. For the L‐dc‐Au‐based NRs, the regions with higher HE Rate under LCP exhibits more than those under RCP (Figure S24), the opposite is the case for D‐dc‐Au‐based NRs, indicating the selective interaction between the chiral nanostructures and CPL. Therefore, L‐dc‐Au‐based NRs can provide more HEs under LCP than RCP, which affects the rate of the catalytic reaction. The corresponding results are also reflected in ΔHE Rate. For the da‐Au‐based NRs, the ΔHE rate value of Au NRs is only approximately 0.1, which is much smaller than that of L‐dc/D‐dc‐Au NRs. Therefore, the influence of Au NRs on chiral HEs is very small here. The ΔHE rate of the Ag shell can reach approximately 0.6, and there are significant differences between the L‐dc‐and the D‐dc‐NRs. Therefore, for the nanostructures based on da‐Au, the Ag shell plays the main role in the catalytic process. Photocurrent measurements under CPL further reveal that the dc‐Au@c‐Ag NRs exhibit a strong handedness‐dependent photocurrent: D‐dc‐Au@L‐Ag NRs outperform under RCP compared to LCP, while D‐dc‐Au@D‐Ag NRs show better performance under LCP than under RCP (Figure S27). The bare D‐dc‐Au NRs still respond to both LCP and RCP illumination, but the photocurrent is weak and shows only a very small polarization dependence compared with the Ag‐shelled samples. Therefore, all these observations demonstrate that both the chiral nature of the Ag shell and the Au NRs synergistically govern the HE Rate and ΔHE Rate responses to LCP and RCP. The Ag shell plays a dominant role in chiral hot electron generation, while the chiral Au core modulates the selective interaction with CPL, jointly regulating the catalytic reaction rate associated with hot electrons.

## 3 Conclusion

In summary, we have developed a chiral plasmonic printing strategy that utilizes CPL as a chemically chiral ligand‐free stimulus to drive the asymmetric deposition of silver onto dc‐Au NRs, yielding hierarchical chiral Au core @ chiral Ag semi‐core‐shell structures with programmable chiroptical properties. The synergistic interplay between the intrinsic chirality of the Au core and the handedness of the incident CPL enables precise optical control over the morphology and optical activity of the resulting nanostructures. Importantly, the chiral Ag shell dominates the circular‐polarization‐dependent hot‐electron generation, which in turn governs the circularly polarized photocatalytic activity performance in the model reduction of 4‐nitrophenol. Critically, the experimentally observed circular‐polarization‐dependent and circularly polarized photocatalytic activity performance in the model reduction of 4‐nitrophenol aligns consistently with the theoretically simulated hot‐electron generation profiles, confirming the proposed mechanism of chirality‐directed hot‐carrier generation and transfer. This agreement between experiment and simulation underscores the reliability of our approach in rationally designing chiral plasmonic catalysts, where the handedness of the Ag shell plays a decisive role in steering both optical response and catalytic function. Looking forward, this CPL‐driven printing strategy can be extended to other metal combinations and more complex chiral architectures, paving the way for programmable chiral nanocatalysts and photonic devices. Moreover, the precise coupling between chiral optics and catalytic selectivity demonstrated here offers a generalizable platform for developing tailored chiral nanomaterials for asymmetric synthesis and sensing applications.

## Author Contributions

**Yaxin Cao**: data curation, formal analysis, writing – original draft, writing – review and editing, visualization. **Haibo Zhou**: supervision, writing – review and editing. **Shengshi Fan**: writing – review and editing. **Mengli Wu**: data curation, visualization, writing – review and editing. **Guangchao Zheng**: supervision, conceptualization, investigation, project administration, funding acquisition, writing – original draft, writing – review and editing. **Shenli Wang**: supervision, writing – review and editing, project administration. **Haoyu Li**: software, data curation, writing – original draft, writing – review and editing, visualization.

## Conflicts of Interest

The authors declare no conflicts of interest.

## Supporting information




**Supporting File**: advs76451‐sup‐0001‐SuppMat.docx.

## Data Availability

The data that supports the findings of this study are available in the supplementary material of this article.

## References

[1] J. Kwon , K. H. Park , W. J. Choi , N. A. Kotov , and J. Yeom , “Chiral Spectroscopy of Nanostructures,” Accounts of Chemical Research 56 (2023): 1359–1372, 10.1021/acs.accounts.2c00756. 37256726

[2] A. Lininger , G. Palermo , A. Guglielmelli , et al., “Chirality in Light–Matter Interaction,” Advanced Materials 35 (2023): 2107325, 10.1002/adma.202107325. 35532188

[3] G. Zheng , J. He , V. Kumar , et al., “Discrete Metal Nanoparticles With Plasmonic Chirality,” Chemical Society Reviews 50 (2021): 3738–3754, 10.1039/C9CS00765B. 33586721

[4] M. Hentschel , M. Schäferling , X. Duan , H. Giessen , and N. Liu , “Chiral Plasmonics,” Science Advances 3 (2017): 1602735, 10.1126/sciadv.1602735. PMC5435411 28560336

[5] V. K. Valev , J. J. Baumberg , C. Sibilia , and T. Verbiest , “Chirality and Chiroptical Effects in Plasmonic Nanostructures: Fundamentals, Recent Progress, and Outlook,” Advanced Materials 25 (2013): 2517–2534, 10.1002/adma.201205178. 23553650

[6] S. Wang , X. Liu , S. Mourdikoudis , et al., “Chiral Au Nanorods: Synthesis, Chirality Origin, and Applications,” ACS Nano 16 (2022): 19789–19809, 10.1021/acsnano.2c08145. 36454684

[7] Y. Zhao , J. Xie , Y. Tian , et al., “Colloidal Chiral Carbon Dots: An Emerging System for Chiroptical Applications,” Advanced Science 11 (2024): 2305797, 10.1002/advs.202305797. 38268241 PMC10987166

[8] B. Ni , M. Mychinko , S. Gómez‐Graña , et al., “Chiral Seeded Growth of Gold Nanorods Into Fourfold Twisted Nanoparticles With Plasmonic Optical Activity,” Advanced Materials 36 (2024): 2312066, 10.1002/adma.202312066. 38161223

[9] H.‐E. Lee , H.‐Y. Ahn , J. Mun , et al., “Amino‐Acid‐ And Peptide‐Directed Synthesis Of Chiral Plasmonic Gold Nanoparticles,” Nature 556 (2018): 360–365, 10.1038/s41586-018-0034-1. 29670265

[10] X. Sun , H. Kong , Q. Zhou , et al., “Chiral Plasmonic Nanoparticle Assisted Raman Enantioselective Recognition,” Analytical Chemistry 92 (2020): 8015–8020, 10.1021/acs.analchem.0c01311. 32449359

[11] G. González‐Rubio , J. Mosquera , V. Kumar , et al., “Micelle‐Directed Chiral Seeded Growth On Anisotropic Gold Nanocrystals,” Science 368 (2020): 1472–1477, 10.1126/science.aba0980. 32587018

[12] M. L. Solomon , A. A. E. Saleh , L. V. Poulikakos , J. M. Abendroth , L. F. Tadesse , and J. A. Dionne , “Nanophotonic Platforms for Chiral Sensing and Separation,” Accounts of Chemical Research 53 (2020): 588–598, 10.1021/acs.accounts.9b00460. 31913015

[13] X. Chen , S. Wang , X. Zhang , et al., “Rational Construction Of Enzyme‐Like Stereoselective Magnetic Chiral Nanozymes,” Nano Research 18 (2025): 94907307, 10.26599/NR.2025.94907307.

[14] G. Zheng , Z. Bao , J. Pérez‐Juste , et al., “Tuning the Morphology and Chiroptical Properties of Discrete Gold Nanorods With Amino Acids,” Angewandte Chemie International Edition 57 (2018): 16452–16457, 10.1002/anie.201810693. 30375752

[15] F. Cai , B. Bai , Q. Wu , et al., “Blue Quantum Dot Light‐Emitting Diodes Toward Full‐Color Displays: Materials, Devices, And Large‐Scale Fabrication,” Nano Letters 25 (2025): 1–15, 10.1021/acs.nanolett.4c02968. 39725479

[16] M. Gao , K. Zhang , X.‐T. Hao , and W. Qin , “Synergistic Effect of Chiral Nanofibers Amplifying the Orbit Angular Momentum To Enhance Optomagnetic Coupling,” ACS Nano 16 (2022): 4843–4850, 10.1021/acsnano.2c00404. 35171574

[17] R. C. Devlin , A. Ambrosio , N. A. Rubin , J. P. B. Mueller , and F. Capasso , “Arbitrary Spin‐To–Orbital Angular Momentum Conversion Of Light,” Science 358 (2017): 896–901, 10.1126/science.aao5392. 29097490

[18] K. Saito , Y. Nemoto , and Y. Ishikawa , “Circularly Polarized Light‐Induced Chiral Growth Of Achiral Plasmonic Nanoparticles Dispersed In A Solution,” Nano Letters 24 (2024): 12840–12848, 10.1021/acs.nanolett.4c03183. 39356044

[19] L. Xu , X. Wang , W. Wang , et al., “Enantiomer‐Dependent Immunological Response To Chiral Nanoparticles,” Nature 601 (2022): 366–373, 10.1038/s41586-021-04243-2. 35046606

[20] T. Qiao , P. Bordoloi , A. E. Ashworth , et al., “Increasing the Structural Chirality of Metal Nanocrystals Created by Circularly Polarized Light via Surface Ligand Engineering,” Small 21 (2025): 2502440, 10.1002/smll.202502440. 40528545

[21] N. Ichiji , T. Ishida , I. Morichika , D. Oue , T. Tatsuma , and S. Ashihara , “Selective Enhancement of the Optical Chirality and Spin Angular Momentum in Plasmonic Near‐Fields,” Nano Letters 25 (2025): 12578–12584, 10.1021/acs.nanolett.5c02776. 40788763 PMC12372797

[22] E. Efrati and W. T. M. Irvine , “Orientation‐Dependent Handedness And Chiral Design,” Physical Review X 4 (2014): 011003, 10.1103/PhysRevX.4.011003.

[23] J. Y. Kim , C. McGlothin , M. Cha , et al., “Direct‐Write 3D Printing Of Plasmonic Nanohelicoids By Circularly Polarized Light,” Proceedings of the National Academy of Sciences 121 (2024): 2312082121, 10.1073/pnas.2312082121. PMC10945859 38446854

[24] Y. Zhang , Y. Sun , X. Ren , et al., “Chiral Polar Bifunctional Polyimide Enantiomers for Asymmetric Photo‐ and Piezo‐Catalysis,” Angewandte Chemie International Edition 64 (2025): 202416221, 10.1002/anie.202416221. 39370777

[25] S. Li , X. Xu , L. Xu , H. Lin , H. Kuang , and C. Xu , “Emerging Trends In Chiral Inorganic Nanomaterials For Enantioselective Catalysis,” Nature Communications 15 (2024): 3506, 10.1038/s41467-024-47657-y. PMC11045795 38664409

[26] Q. Wang , J. Liu , S. Li , et al., “Optical Switching of Catalytic Pathways for Hydrogen Generation via Light‐Handedness Control on Chiral Nanostructures,” Angewandte Chemie International Edition 64 (2025): 202517047, 10.1002/anie.202517047. 41131912

[27] F. Wang , W. Yang , Q. Ding , et al., “Chiral Au@CeO_2_ Helical Nanorods With Spatially Separated Structures for Polarization‐Dependent N_2_ Photofixation,” Angewandte Chemie International Edition 64 (2025): 202415031, 10.1002/anie.202415031. 39320103

[28] P. Bainova , J.‐P. Joly , M. Urbanova , et al., “Plasmon‐Assisted Chemistry Using Chiral Gold Helicoids: Toward Asymmetric Organic Catalysis,” ACS Catalysis 13 (2023): 12859–12867, 10.1021/acscatal.3c02958.

[29] T. Luo , H. Li , Z. Zhang , et al., “Super‐Heterostructures of Twisted Pd Nanoarrays Epitaxially Grown on Chiral Au Nanorods Boost Circularly Polarized Photocatalysis,” Advanced Science 12 (2025): 2502848, 10.1002/advs.202502848. 40270460 PMC12245117

[30] H. Lee , Y. S. Park , E. Kwon , et al., “Cu_2_O/Cu Chiral Catalysts for Highly Selective Solar‐Assisted CO_2_ ‐to‐CO Electroreduction,” Advanced Functional Materials 35 (2025): 08577, 10.1002/adfm.202508577.

[31] W. Fu , Q. Gao , C. Zhang , et al., “Exploring Geometric Chirality in Nanocrystals for Boosting Solar‐to‐Hydrogen Conversion,” Angewandte Chemie International Edition 63 (2024): 202411871, 10.1002/anie.202411871. 39054405

[32] H. Kang , D.‐I. Won , H. J. Lee , et al., “Polarization‐Selective Efficient Hydrogen Evolution Reactions via Chiral Photocatalysis,” Advanced Materials 38 (2026): 20438, 10.1002/adma.73255. 41540632

[33] G. Zheng , S. Jiao , W. Zhang , et al., “Fine‐Tune Chiroptical Activity In Discrete Chiral Au Nanorods,” Nano Research 15 (2022): 6574–6581, 10.1007/s12274-022-4212-y.

[34] M. Ghalawat , D. Feferman , L. V. Besteiro , et al., “Chiral Symmetry Breaking in Colloidal Metal Nanoparticle Solutions by Circularly Polarized Light,” ACS Nano 18 (2024): 28279–28291, 10.1021/acsnano.4c09349. 39367853 PMC11483945

[35] P. Lyu , R. Espinoza , and S. C. Nguyen , “Photocatalysis of Metallic Nanoparticles: Interband vs Intraband Induced Mechanisms,” The Journal of Physical Chemistry C 127 (2023): 15685–15698, 10.1021/acs.jpcc.3c04436. PMC10440817 37609384

[36] S. Lee , C. Fan , A. Movsesyan , et al., “Unraveling the Chirality Transfer From Circularly Polarized Light to Single Plasmonic Nanoparticles,” Angewandte Chemie International Edition 63 (2024): 202319920, 10.1002/anie.202319920. 38236010

[37] W. Fu , Q. Gao , C. Zhang , et al., “Exploring Geometric Chirality in Nanocrystals for Boosting Solar‐to‐Hydrogen Conversion,” Angewandte Chemie International Edition 63 (2024): 202411871, 10.1002/anie.202411871. 39054405

[38] Y. Jin , W. Fu , Z. Wen , et al., “Chirality Engineering of Colloidal Copper Oxide Nanostructures for Tailored Spin‐Polarized Catalysis,” Journal of the American Chemical Society 146 (2024): 2798–2804, 10.1021/jacs.3c12965. 38145451

[39] Y. Negrín‐Montecelo , A. Movsesyan , J. Gao , et al., “Chiral Generation of Hot Carriers for Polarization‐Sensitive Plasmonic Photocatalysis,” Journal of the American Chemical Society 144 (2022): 1663–1671, 10.1021/jacs.1c10526. 35073069

[40] T. Liu , L. V. Besteiro , T. Liedl , M. A. Correa‐Duarte , Z. Wang , and A. O. Govorov , “Chiral Plasmonic Nanocrystals for Generation of Hot Electrons: Toward Polarization‐Sensitive Photochemistry,” Nano Letters 19 (2019): 1395–1407, 10.1021/acs.nanolett.8b05179. 30681343

[41] L. V. Besteiro , X.‐T. Kong , Z. Wang , G. Hartland , and A. O. Govorov , “Understanding Hot‐Electron Generation and Plasmon Relaxation in Metal Nanocrystals: Quantum and Classical Mechanisms,” ACS Photonics 4 (2017): 2759–2781, 10.1021/acsphotonics.7b00751.

