# Supplementary material for: Chiral Plasmonic Printing of Discrete Hierarchical Chiral Au@Chiral Ag Semi‐Core‐Shell Nanorods With Chiral Plasmon‐Enhanced Photocatalysis
Source: Adv Sci (Weinh). 2026 Jul 20:e76451. Online ahead of print. doi: 10.1002/advs.76451 (PMC13384036; doi:10.1002/advs.76451)
Supplement: Supplementary file 1 — Supporting File: advs76451‐sup‐0001‐SuppMat.docx. [file ADVS-9999-e76451-s001.docx]

**Supporting Information**

**Chiral Plasmonic Printing of** **Discrete Hierarchical Chiral Au@Chiral Ag Semi-Core-Shell Nanorods With Chiral plasmon-Enhanced Photocatalysis**

Yaxin Cao#, Haoyu Li#, Mengli Wu,# Shengshi Fan, Shenli Wang*, Haibo Zhou,* Guangchao Zheng*

X. Cao, H. Li, M. Wu, S. Fan, G. Zheng

Colloidal Physics Group, Key Laboratory of Materials Physics, Ministry of Education, School of Physics and Laboratory of Zhongyuan Light, Zhengzhou University, Zhengzhou 450001, P. R. China. E-mail: [gczheng@zzu.edu.cn](mailto:gczheng@zzu.edu.cn)

G. Zheng

Institute of Quantum Materials and Physics, Henan Academy of Sciences, Zhengzhou 450046, P. R. China

S. Wang

College of Food Science and Technology, Henan University of Technology, Zhengzhou 450001, P. R. China. Email: [wangshenli@126.com](mailto:wangshenli@126.com)

H. Zhou

Institute of Pharmaceutical Analysis, College of Pharmacy, Jinan University, Guangzhou 510632, China.

Email: [haibo_zhou365@163.com](mailto:haibo_zhou365@163.com)

**Materials**

Hexadecyltrimethylammonium bromide (CTAB, >99%), sodium oleate (NaOL, >97%), hexadecyltrimethylammonium chloride (CTAC, >99%), silver nitrate (AgNO_3_, >99%), L/D-Cysteine (L/D-Cys), L-ascorbic acid (AA, >99.5%), hydrogen tetrachloroaurate trihydrate (HAuCl_4_·3H_2_O) sodium borohydride (NaBH_4_, 99%), trisodium citrate dihydrate (citNa), and Poly (sodium 4-styrenesulfonate) (PSS, Mw ~70,000) were purchased from Adamas. Ultrapure water was supplied from a Milli-Q water (18.25 MΩ) purification system. All chemical reagents were used for synthesis as received without further purification.

**Synthesis of discrete achiral Au nanorods** **(da-Au NRs)**

A seed solution was prepared by adding an aqueous HAuCl_4_ solution (0.25 mL, 10 mM) into CTAB solution (5 mL, 0.2 M) in a 10 mL vial. Then, 0.6 mL of fresh NaBH_4_ (0.01 M) was diluted to 1 mL with water and injected into the mixture under vigorous stirring (800 rpm). After stirring for 2 min, the seed solution was aged at 30 ℃ for 30 min before use.

For the growth solution, CTAB (7 g) and NaOL (1.234 g) were dissolved in 250 mL of water (≈60  ℃) in a 1 L beaker. After cooling to room temperature, AgNO_3_ solution (18 mL, 4 mM) was added, and the mixture was kept undisturbed at 30   ℃ for 15 min. Next, HAuCl_4_ solution (250 mL, 1 mM) was introduced. After standing undisturbed for 90 min, the solution turned colorless, followed by the addition of HCl (1.5 mL, 12 M) to adjust the pH. The solution was left undisturbed for another 15 min, after which ascorbic acid (AA, 1.25 mL, 0.064 M) was added under vigorous stirring for 30 s. Finally, the seed solution (0.4 mL) was injected into the growth solution. The resulting mixture was stirred for 30 s and then kept static at 30  ℃ for 12 h to allow the growth of Au NRs. The resulting nanoparticles were collected by centrifugation twice (7000 rpm, 10 min each) and redispersed in 80 mM CTAC for further use.

**Synthesis of discrete chiral**-**Au NRs (dc**-**Au NRs)**

In a 10 mL vial, 4 mL of 40 mM CTAC solution was mixed with 200 μL of 1 mM HAuCl_4_ under stirring for 30 s. Subsequently, 475 μL of 0.1 M AA was added, followed by another 30 s of stirring. Then, 20 μL of 10^-5^ M L- or D-cysteine (L-Cys for L-dc-Au NRs, D-Cys for D-dc-Au NRs) and 200 μL of Au NR seed solution were sequentially introduced. Finally, 100 μL of 10 mM HAuCl_4_ was added, and the mixture was left undisturbed at room temperature for 2 h. The resulting nanoparticles were collected by centrifugation twice (4000 rpm, 5  min each) and redispersed in 5 mL of water for further use.

**Synthesis of discrete hierarchical chiral Au core@chiral Ag semi-core-shell NRs (dc-Au @c-Ag NRs) under circularly polarized light (CPL) illumination**

The 10 mL dc-Au NRs were centrifuged, and the precipitate was redispersed in a 10 mL poly(sodium 4-styrenesulfonate) (PSS, 0.4 wt%) solution for 6 h. The PSS-capped Au NRs were then washed twice via centrifugation and redispersed in 10 mL of water. To 10 mL of this colloidal solution, 0.1 mL of sodium citrate (0.1 M) and 0.1 mL of AgNO_3_ (0.1 M) were added. The mixture was subsequently irradiated with left- or right-handed CPL, generated by passing purple LED light through a linear polarizer and a Fresnel rhomb at an intensity of approximately 2.83 mW/cm^2^. Following the photochemical deposition of Ag, the nanoparticles were collected by centrifugation and redispersed in water for further use. The da-Au NRs@c‑Ag nanostructures were synthesized analogously, with the sole modification being the use of discrete achiral (da) Au NRs as seeds.

**Control experiments on the effect of temperature on CPL‑induced Ag growth:** The precipitated dc-Au NRs were redispersed in 10 mL of PSS (0.4 wt%) solution for 6 h. The PSS-capped Au NRs were then washed twice by centrifugation and redispersed in 10 mL of water. To this colloidal solution, 0.1 mL of sodium citrate (0.1 M) and 0.1 mL of AgNO_3_ (0.1 M) were added. The mixture was divided into two equal portions, which were placed in a water bath at 40 °C or 60 °C, respectively, and irradiated with left- or right-handed CPL (405 nm, 2.83 mW/cm²) for 120 min. After Ag deposition, the nanoparticles were collected by centrifugation and redispersed in water.

**CPL‑driven Ag deposition on chiral Au nanorods with exogenously added cysteine:** The 10 mL dc-Au NRs were centrifuged, and the precipitate was redispersed in a 10 mL PSS (0.4 wt%) solution for 6 h. The PSS-capped Au NRs were then washed twice via centrifugation and redispersed in 10 mL of water. To 10 mL of this colloidal solution, 0.1 mL of sodium citrate (0.1 M), 0.1 mL of AgNO_3_ (0.1 M), and 20 µL of cysteine (10⁻⁵ M) were added. The mixture was subsequently irradiated with left- or right-handed CPL, generated by passing purple LED light through a linear polarizer and a Fresnel rhomb at an intensity of approximately 2.83 mW/cm². Following the photochemical deposition of Ag, the nanoparticles were collected by centrifugation and redispersed in water for further use.

**Control experiment without Au NRs under CPL illumination:** For the control reaction without Au NRs, the synthesis procedure was identical to that described above, with the only modification being the replacement of the 10 mL dc-Au NRs colloidal solution with an equal volume of deionized water. Briefly, 10 mL of water was directly mixed with 0.1 mL of sodium citrate (0.1 M) and 0.1 mL of AgNO_3_ (0.1 M), without the need for PSS ligand exchange and washing steps required for Au NRs. The mixture was subsequently irradiated with left- or right-handed CPL, generated by passing purple LED light through a linear polarizer and a Fresnel rhomb at an intensity of approximately 2.83 mW/cm². Following the photochemical reaction, the resulting solution was collected for further characterization.

**Polarized Photocatalysis: Polarized Photocatalysis:** To evaluate the circularly polarized photocatalytic performance of da‑Au@c‑Ag NRs and dc‑Au@c‑Ag NRs, three types of polarized light were tested: linearly polarized light (LP), left circularly polarized light (LCP), and right circularly polarized light (RCP). A laser beam (wavelength: 633 nm, initial power: 100 mW, beam shape: 10 × 10 mm^2^) was converted into LP, LCP, and RCP light, each with a power of 20 mW, by passing it through a linear polarizer and a quarter‑wave plate (λ/4 polarizer, 10 × 10 mm^2^ area, 2 nm thickness), respectively.

To further investigate polarized photocatalysis on 4‑nitrophenol (4‑NP), 0.1 mL of 10 mM 4‑NP and 0.1 mL of 0.1 M NaBH_4_ were mixed with 1.8 mL of water in a vial. Subsequently, 100 μL of either da‑Au@c‑Ag NRs or dc‑Au@c‑Ag NRs was added to the solution. The resulting mixture was then separately illuminated with the different types of polarized light. UV‑vis absorption spectra of the solution were recorded immediately at specified time intervals. All experiments were conducted under various lighting conditions (dark, LP, RCP, LCP) at a constant temperature of 25 ºC. For cycling experiments, the photocatalysts were recovered from the reaction solution by centrifugation and reused.

**Hot electron photocurrent measurements:** The hot electron photocurrent measurements were performed using the glassy carbon electrode. The photoanode was prepared by depositing the nanoparticles on the glassy carbon electrode substrate. The particle densities of D-dc-Au@Ag NRs on the substrate were carefully adjusted to be appropriately equal. Na_2_SO_4_ electrolyte (0.1 M) were sandwiched between the substrate and a cover glass slide, and Pt wire was used as the counter electrode. A supercontinuum laser filtered at 633 nm was employed for illuminating the glassy carbon electrode. The photocurrent of the cell was measured by chopping the laser illumination. The laser spot area is about 28.26 mm^2^, and the power density is about 0.071 W cm^-2^. The hot electron photocurrent was recorded by a Keithley digital source meter.

**Fabrication of dc-Au @SiO_2_ NRs:** 20 mL of dc-Au NRs was concentrated to 10 mL. Subsequently, 3 mL of the concentrated dispersion was mixed with 60 µL of tetraethyl orthosilicate (TEOS) solution and 130 µL of 0.1 M NaOH solution. The reaction mixture was incubated at 45 °C overnight, then centrifuged at 3500 rpm for 5 min twice, and the precipitate was finally dispersed and stored in ethanol.


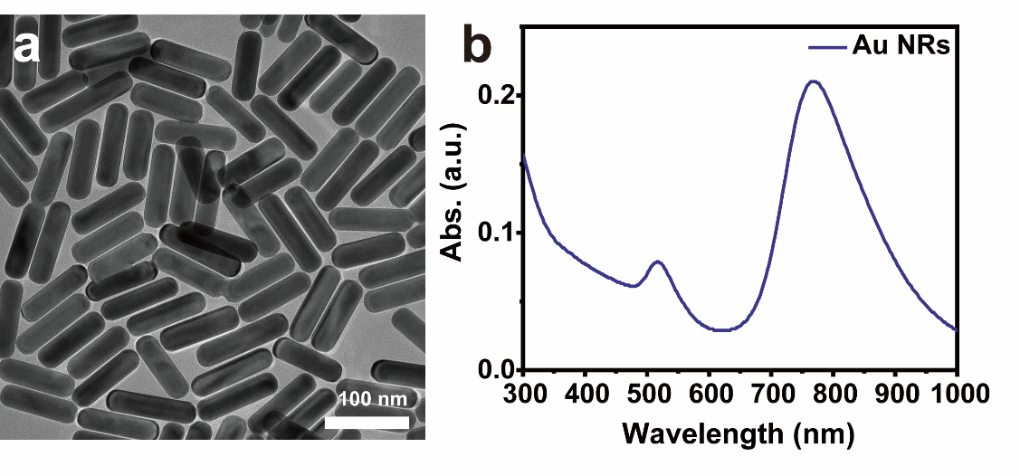


**Figure S1.** (a) TEM images of da-Au NRs. (b) UV-Vis-NIR absorption spectra of da-Au NRs.


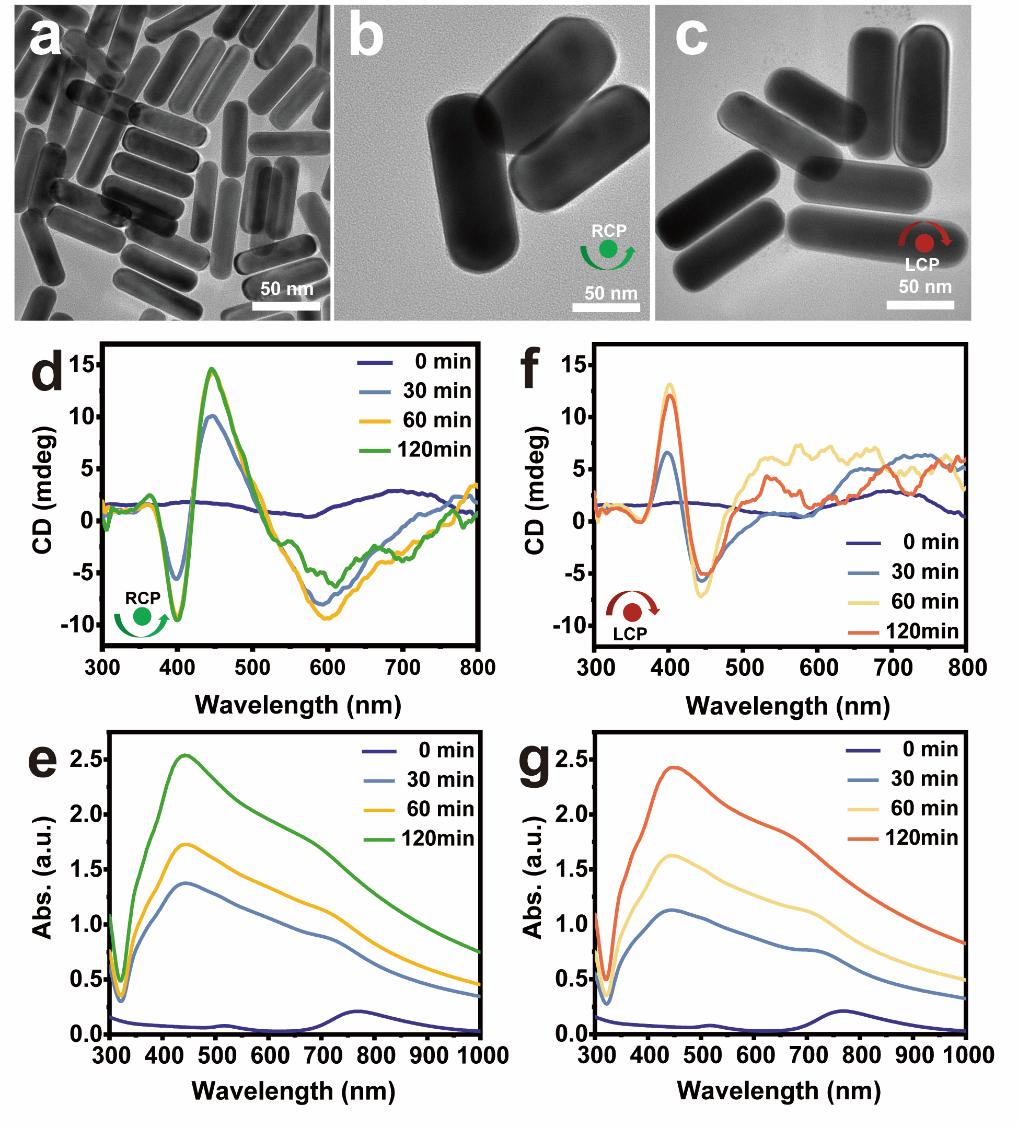


**Figure S2.** (a) TEM of da-Au NRs; da-Au@c-Ag NRs after RCP (b) and LCP (c), respectively. CD and absorbance spectra of da-Au@c-Ag after 120 min 405 nm CPL illumination. (d, e) da-Au@D-Ag under RCP; (f, g) da-Au@L-Ag under LCP. Note: 0 min denotes pre- illumination initial state; Power density of 405 nm: 2.83 mW/cm^2^.


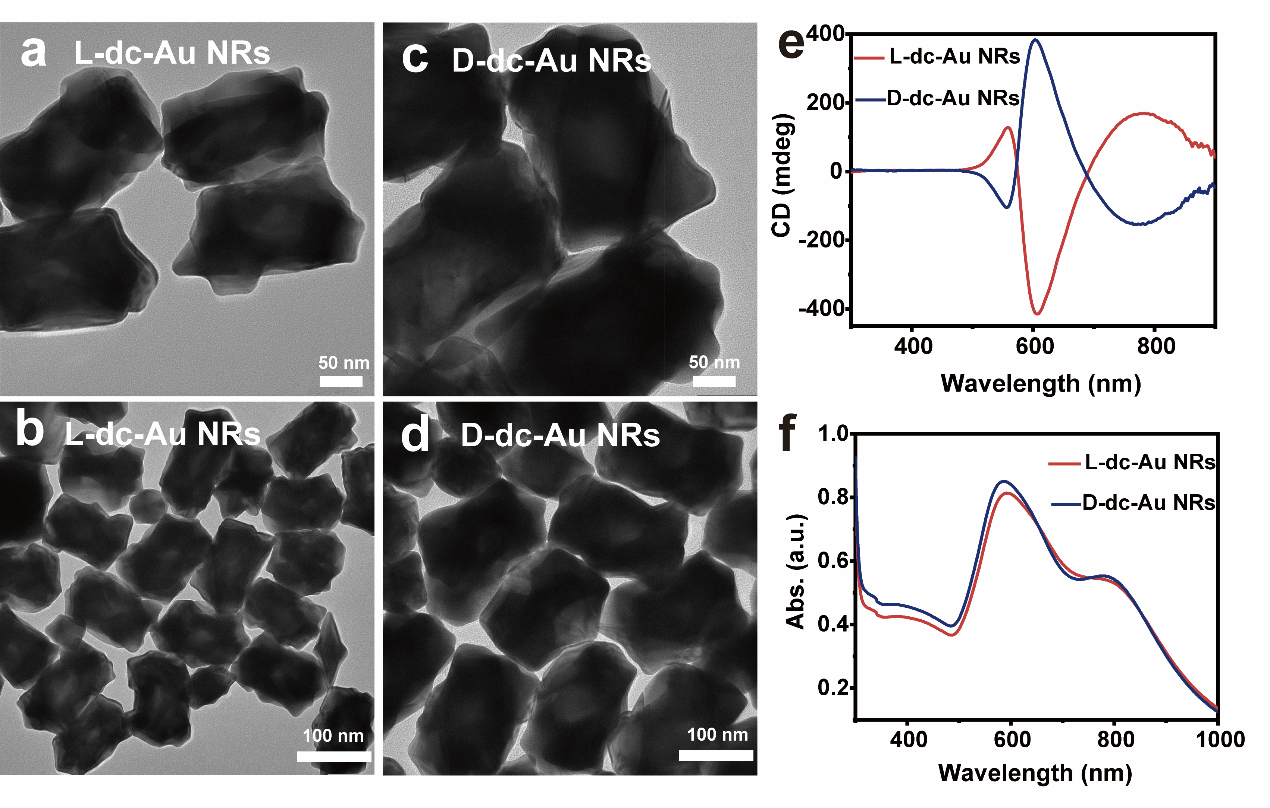


**Figure S3.** TEM images of L-dc-Au NRs (a, b) and D-dc-Au NRs (c, d) with different magnification; (e) CD spectrum of L-dc-Au NRs and D-dc-Au NRs. (f) UV-vis-NIR absorption spectra of L-dc-Au NRs and D-dc-Au NRs.


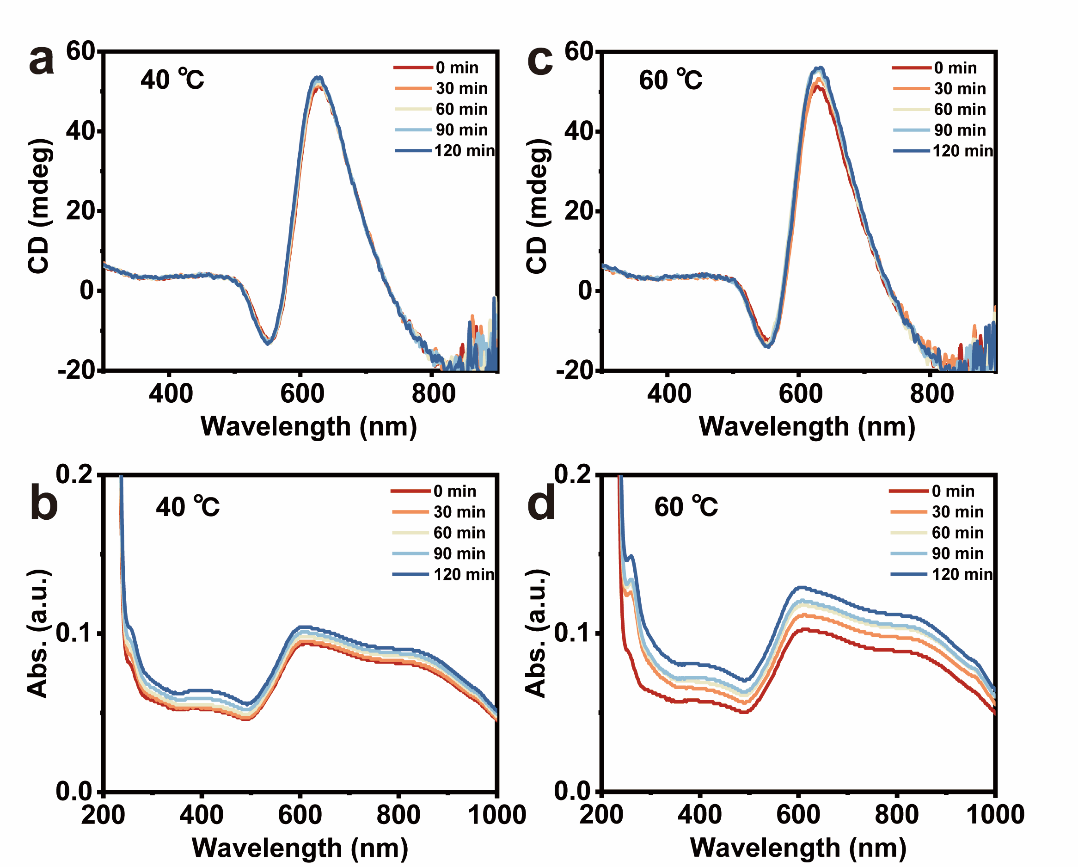


**Figure S4.** Time-dependent CD (a and c) and absorbance (b and d) spectra of Ag deposition on D-dc-Au NRs in the absence of light over 120 min at different temperatures. (a and b) at 40 °C, (c and d) at 60 °C.


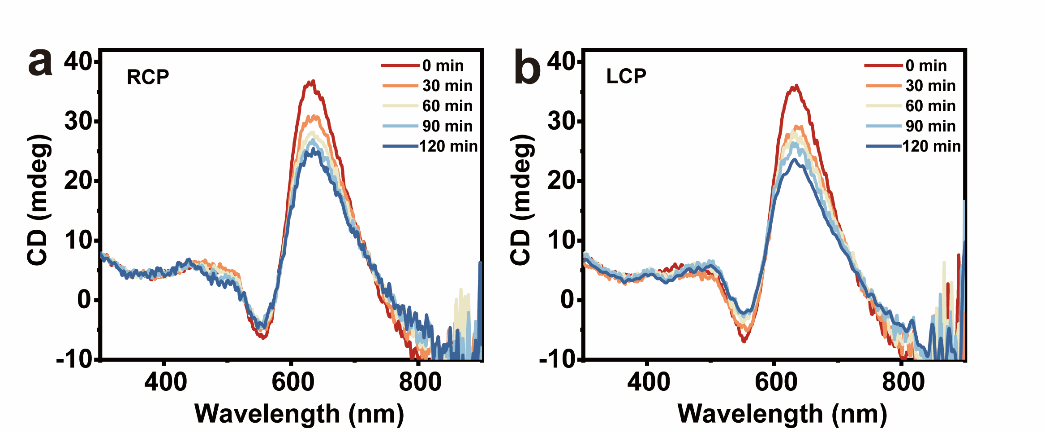


**Figure S5.** Time-dependent CD spectroscopy of Ag deposition on D-dc-Au NRs with additional D-Cys (20 µL of 10⁻⁵ M) under illumination with RCP (a) and LCP (b) over a period of 120 min, respectively.


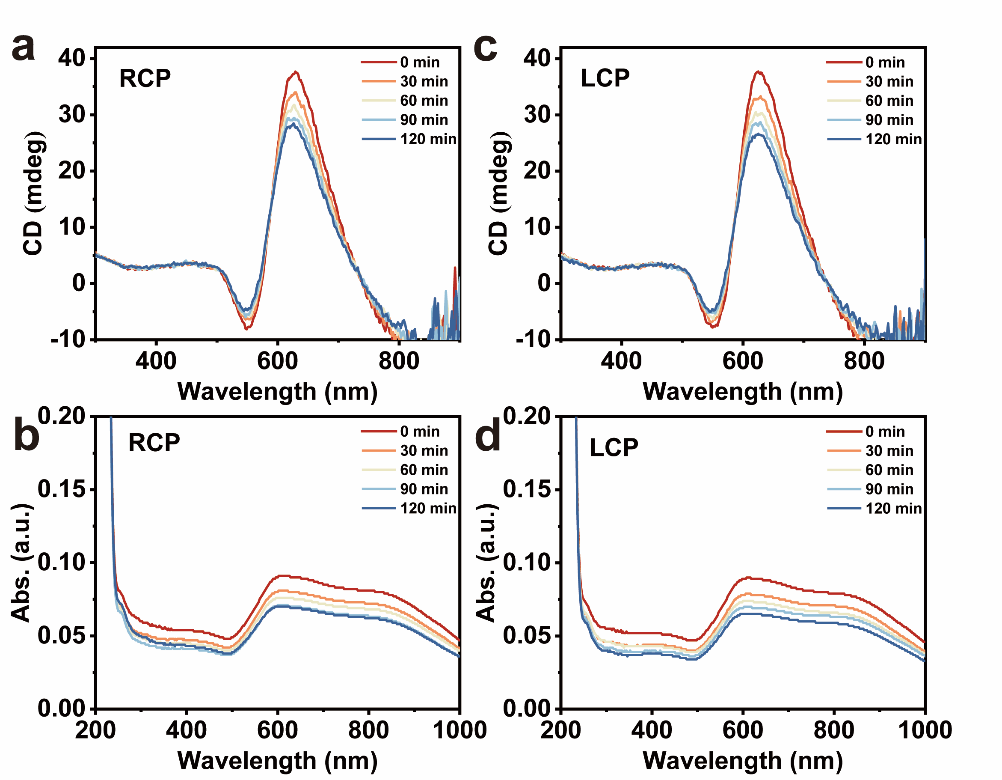


**Figure S6.** Time-dependent CD (a and c) and absorbance (b and d) spectra of the reaction system on D-dc-Au NRs under 633 nm CPL illumination, respectively. (a and b) under RCP; (c and d) under LCP.


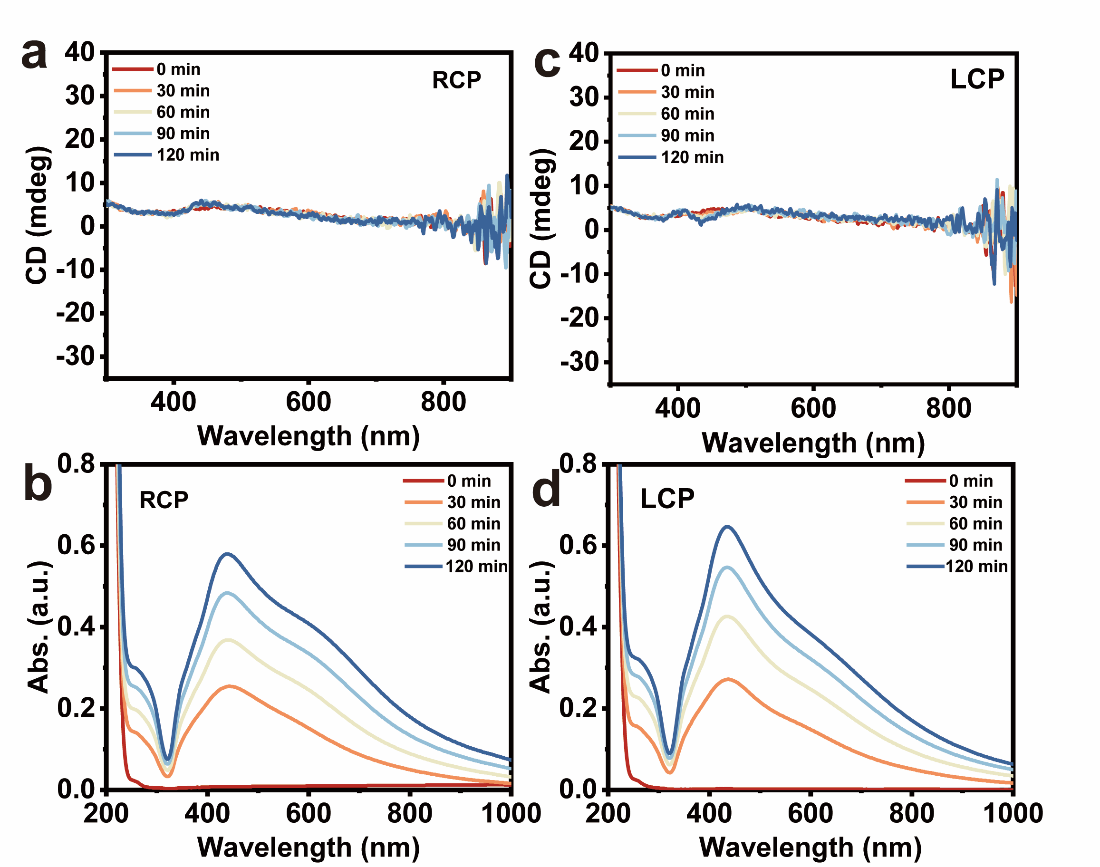


**Figure S7.** Time-dependent CD (a and c) and absorbance (b and d) spectra of the reaction system on D-dc-Au NRs under 405 nm CPL illumination, respectively. (a and b) under RCP; (c and d) under LCP.


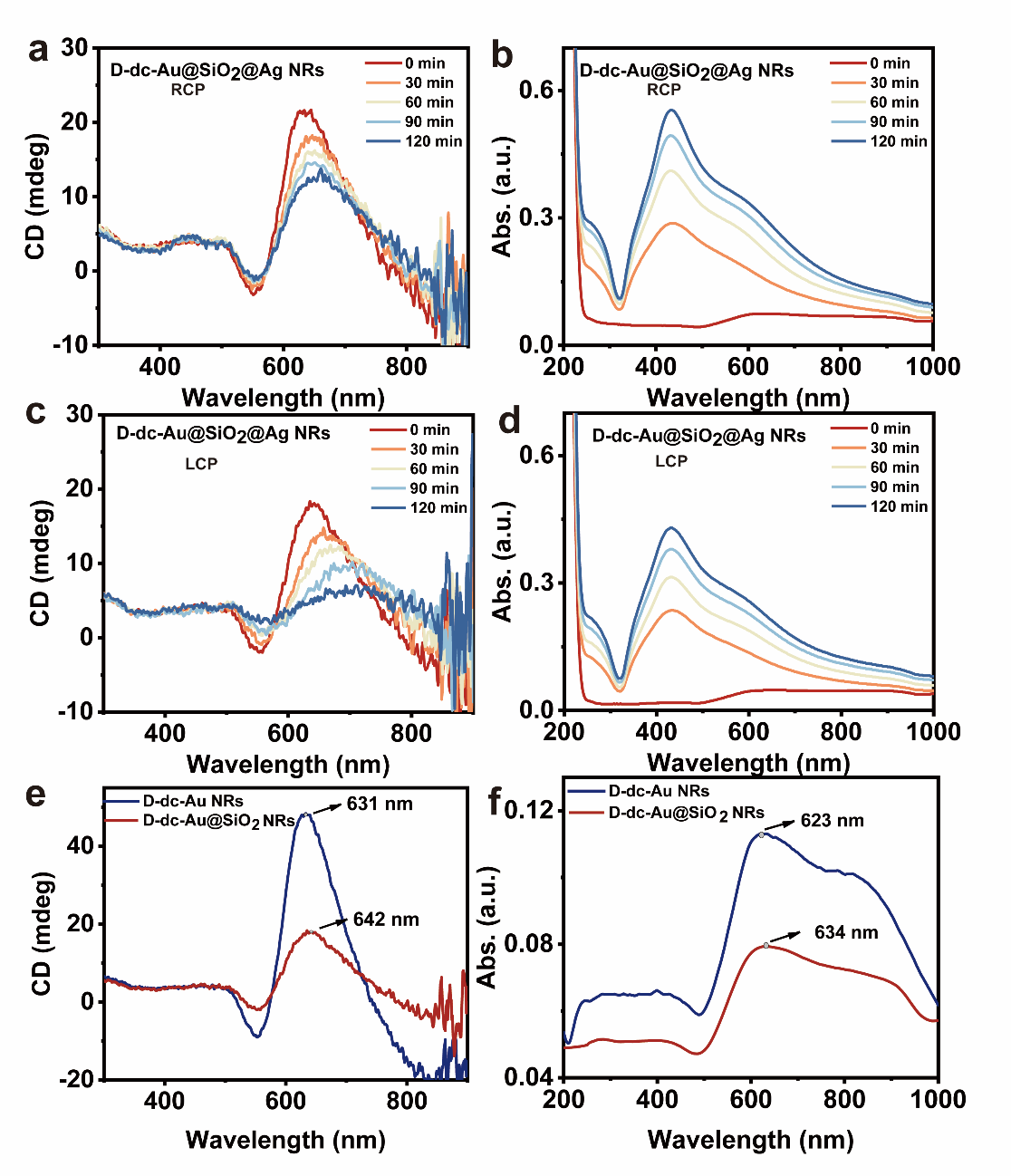


**Figure S8.** Time-dependent CD ((a) RCP, (c) LCP) and extinction ((b) RCP, (d) LCP) spectra of D-dc-Au@SiO_2_ NRs during Ag deposition under CPL illumination. (e) CD and (f) extinction spectra comparing bare and SiO_2_-coated D-dc-Au NRs.


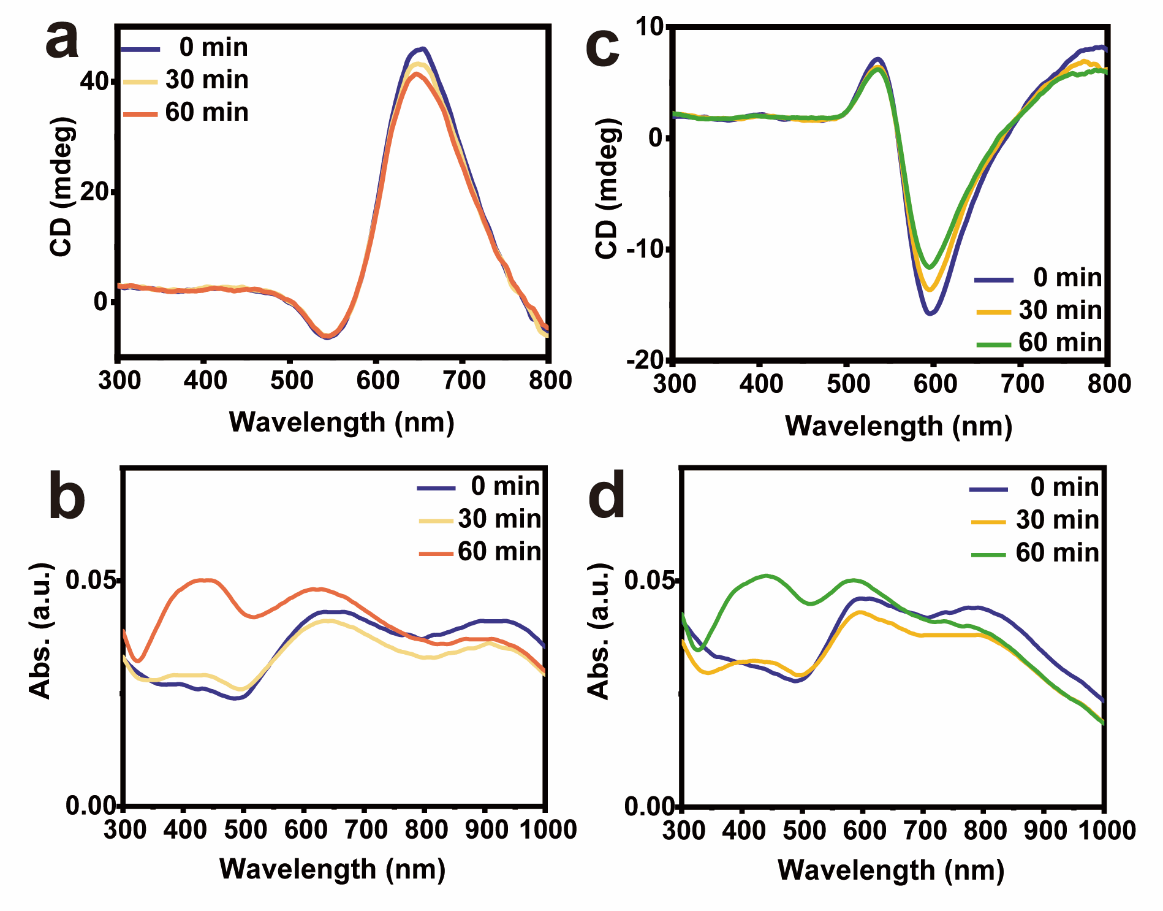


**Figure S9.** CD and absorbance spectra of D-dc-Au@D-Ag (a, b) and L-dc-Au@D-Ag (c, d) NRs before and after 60 min RCP illumination. Power density of 405 nm: 0.226 mW cm^-2^.


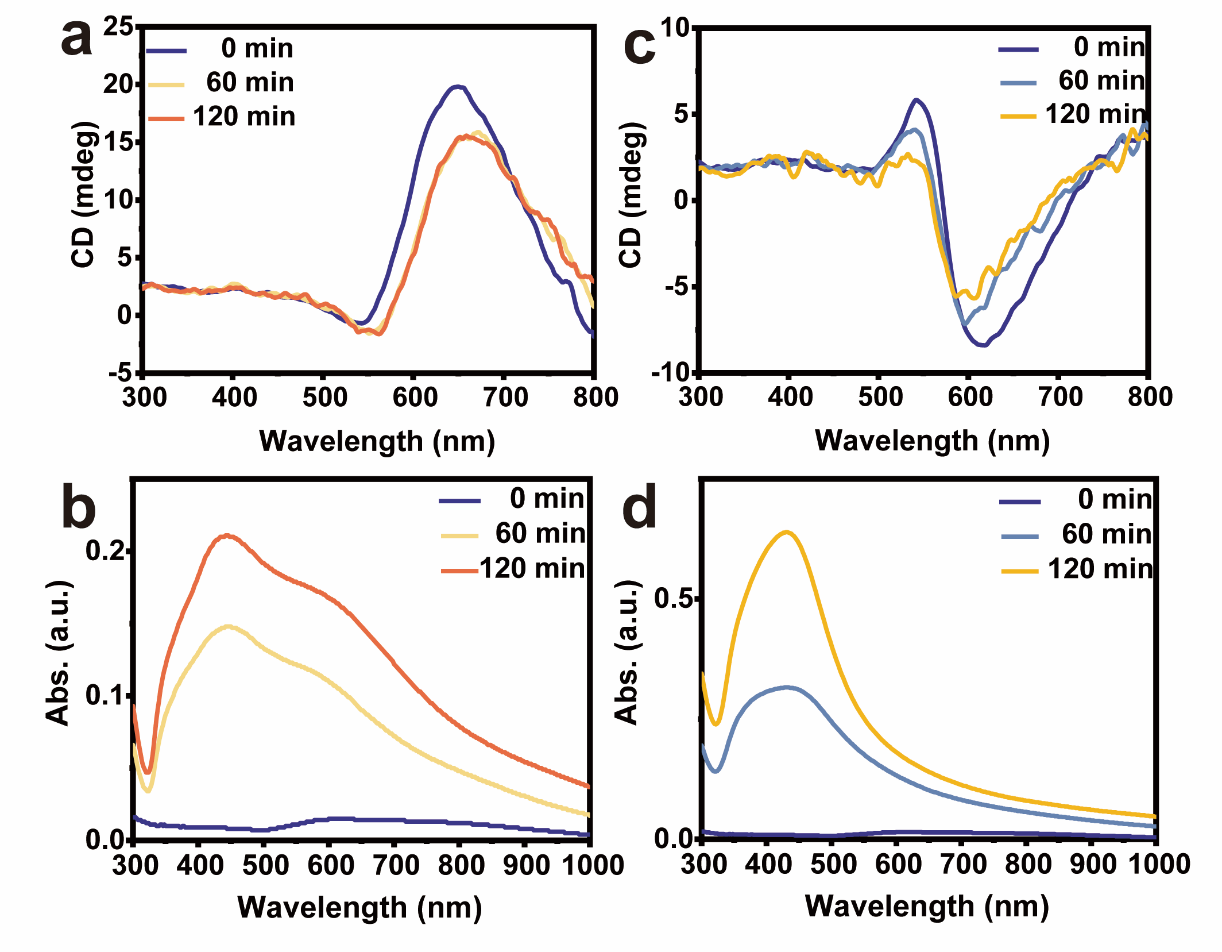


**Figure S10.** CD and absorbance spectra of D-dc-Au@D-Ag (a, b) and L-dc-Au@D-Ag (c, d) NRs before and after 60 min RCP illumination. Power density of 405 nm: 0.453 mW cm^-2^.


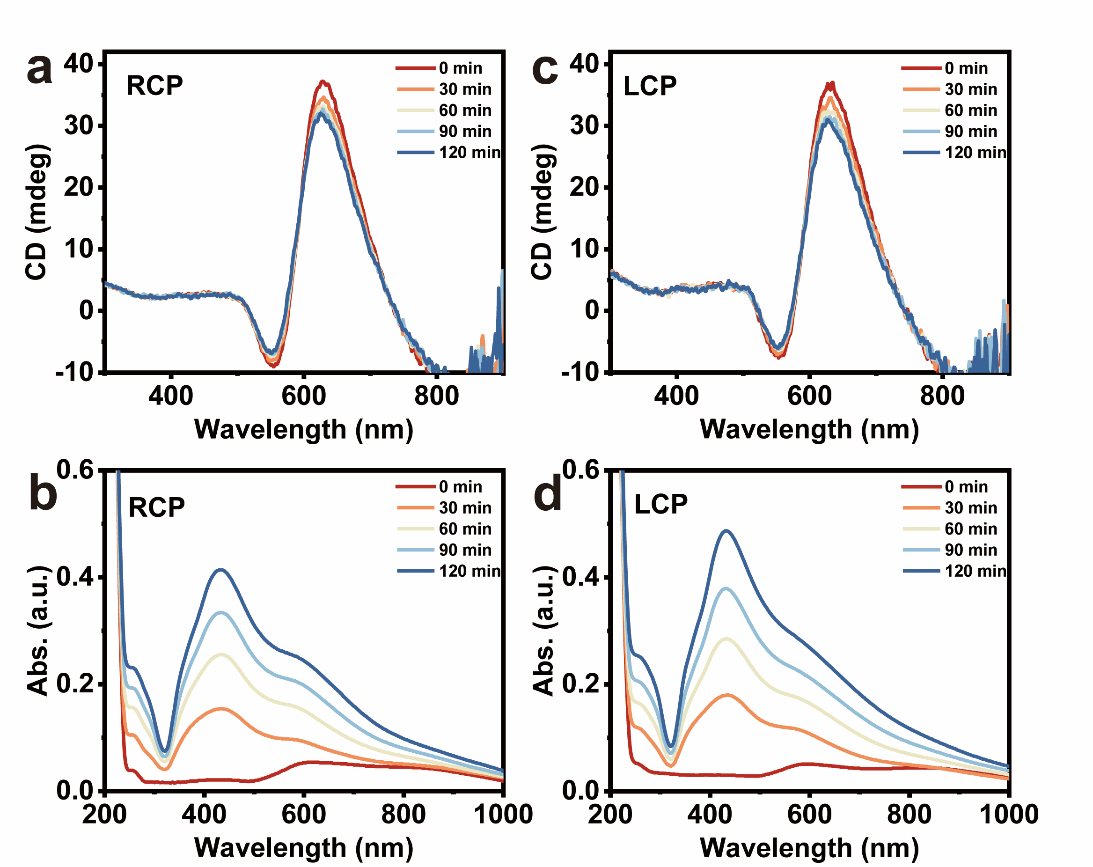


**Figure S11.** Time-dependent CD and UV-vis-NIR absorbance spectra of Ag deposition on D-dc-Au NRs under RCP (a, b) and LCP (c, d) illumination at a power density of 1.41 mW/cm^2^, recorded over a period of 120 min, respectively.


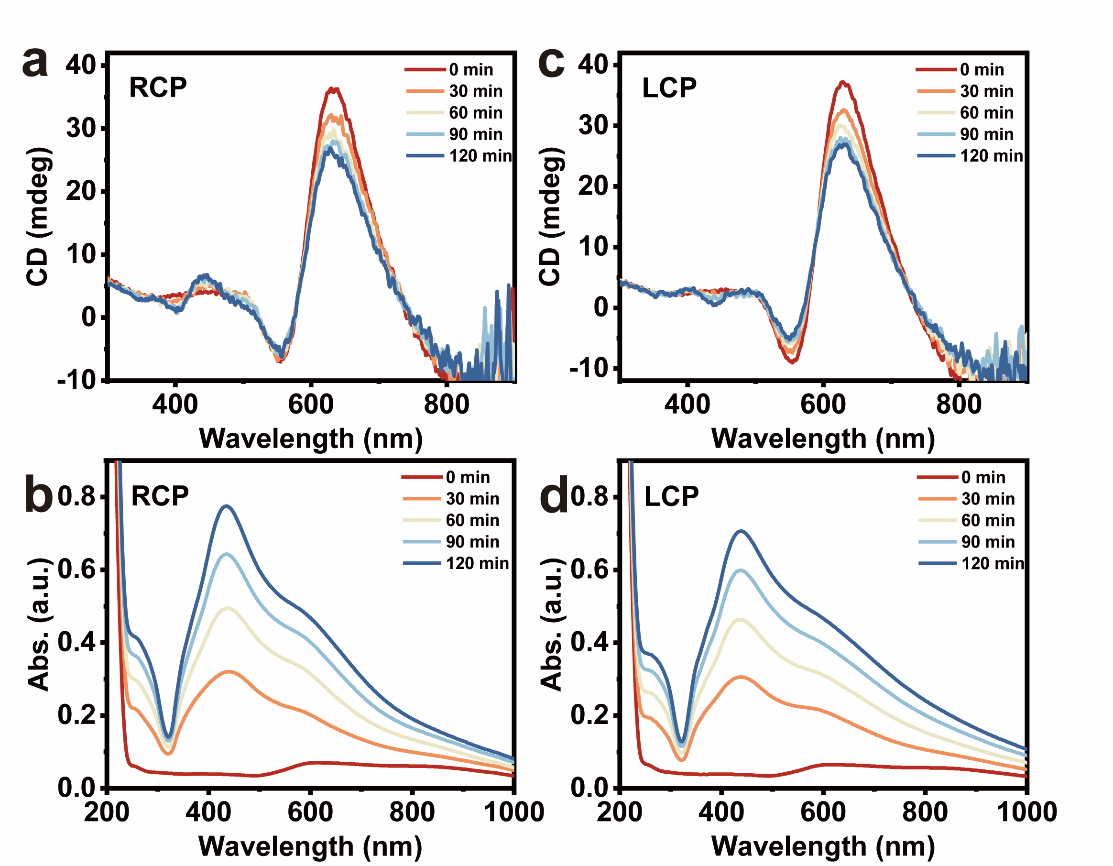


**Figure S12.** Time-dependent CD and UV-vis-NIR absorbance spectra of Ag deposition on D-dc-Au NRs under RCP (a, b) and LCP (c, d) illumination at a power density of 4.24 mW/cm², recorded over a period of 120 min, respectively.


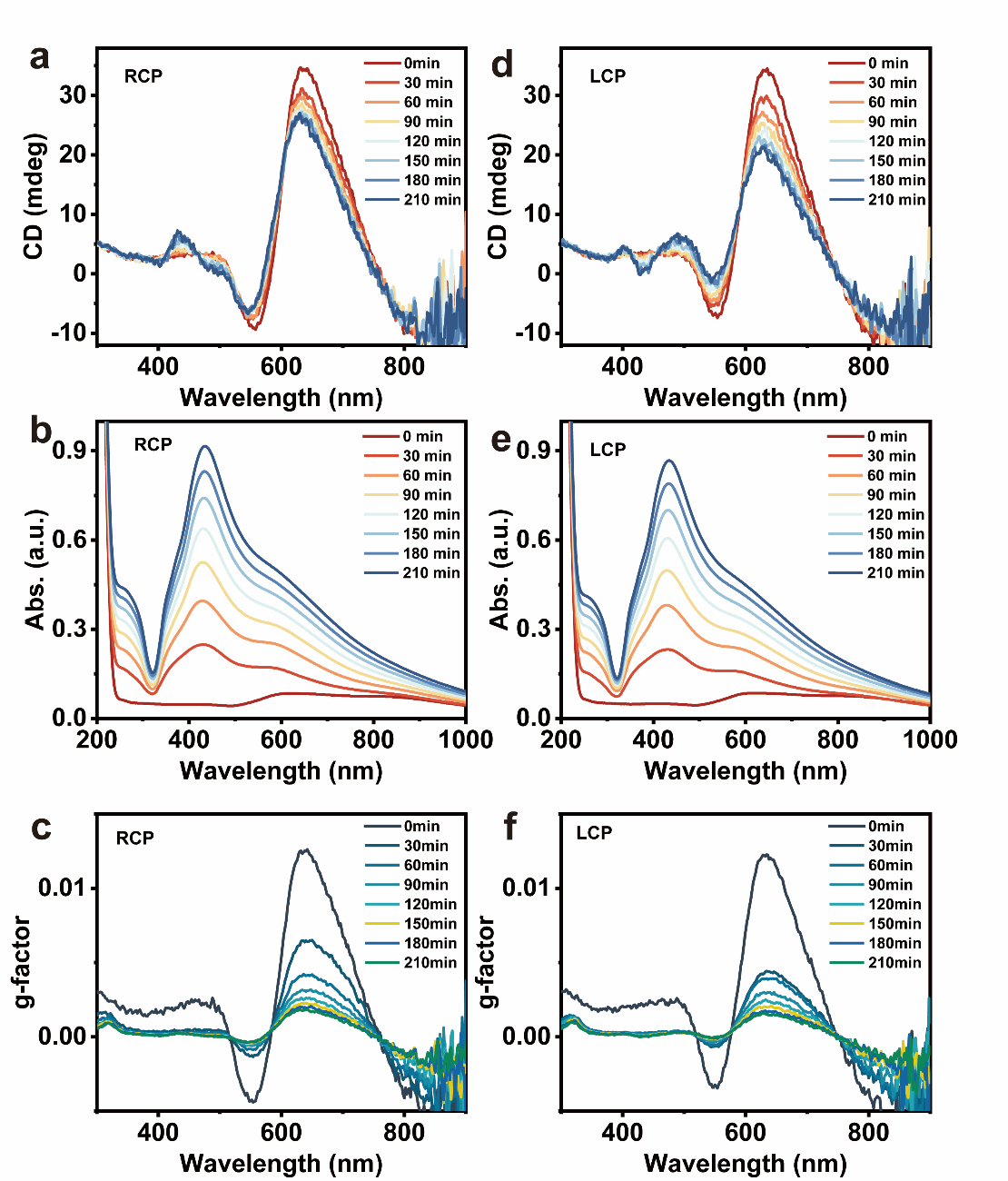


**Figure S13.** Time-dependent CD (a, d), absorbance (b, e), and g-factor (c, f) of D-dc-Au@Ag NRs under RCP (a–c) and LCP (d–f) illumination over 210 min.


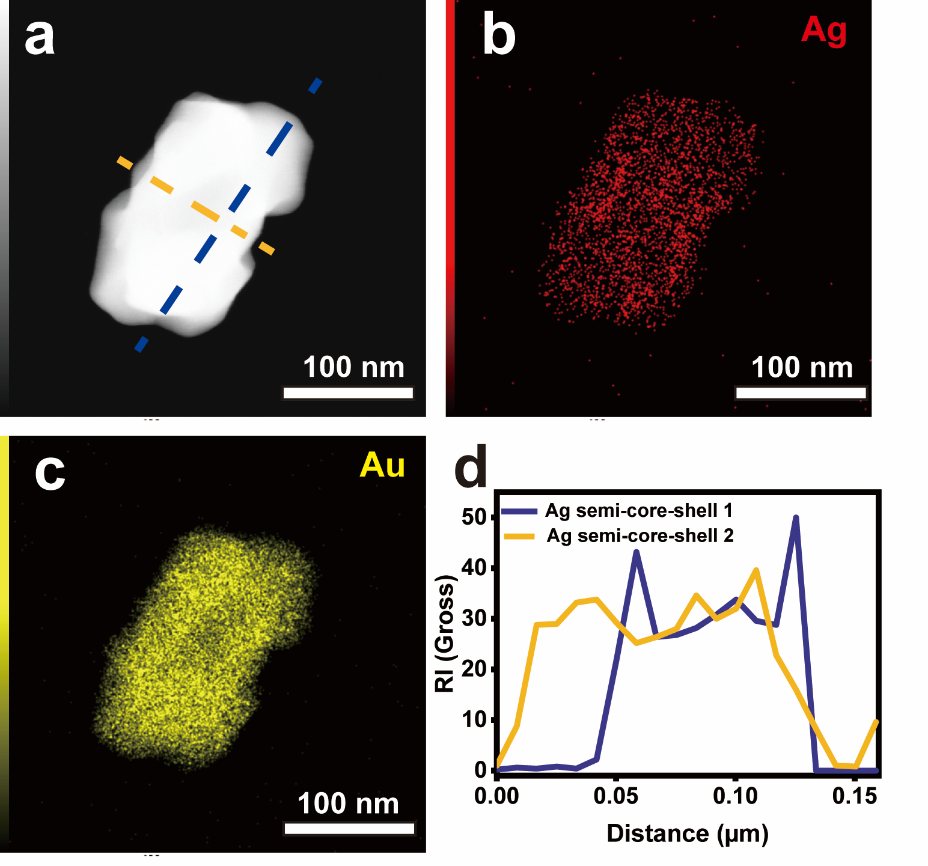


**Figure S14.** (a) HAADF-STEM image of a D-dc-Au@D-Ag NR. (b, c) EDS elemental maps showing the distribution of Ag (b) and Au (c), respectively. (d) HAADF-EDX line-scan profiles of Ag semi-core-shell structures.


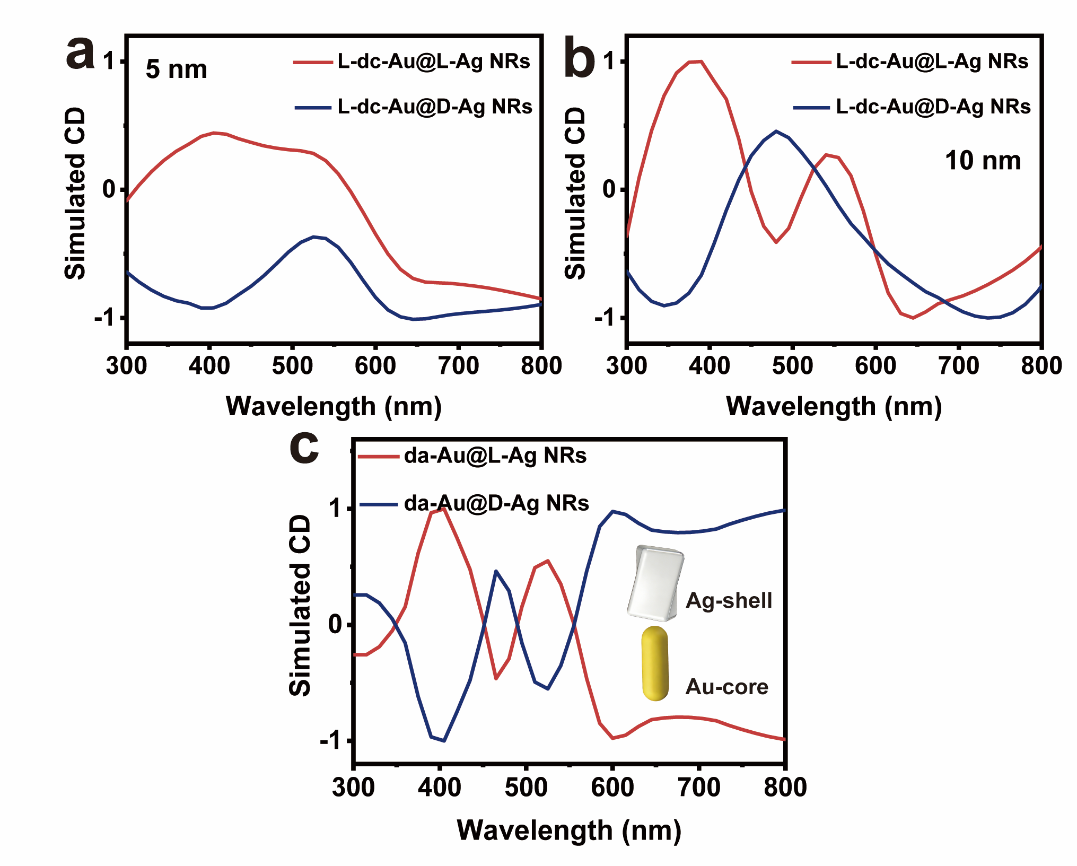


**Figure S15.** Simulated CD spectra of L-dc-Au@c-Ag NRs with Ag semi-core-shell thicknesses of 5 nm (a), 10 nm (b), and 10 nm core-shell da-Au@c-Ag core-shell NRs (c).


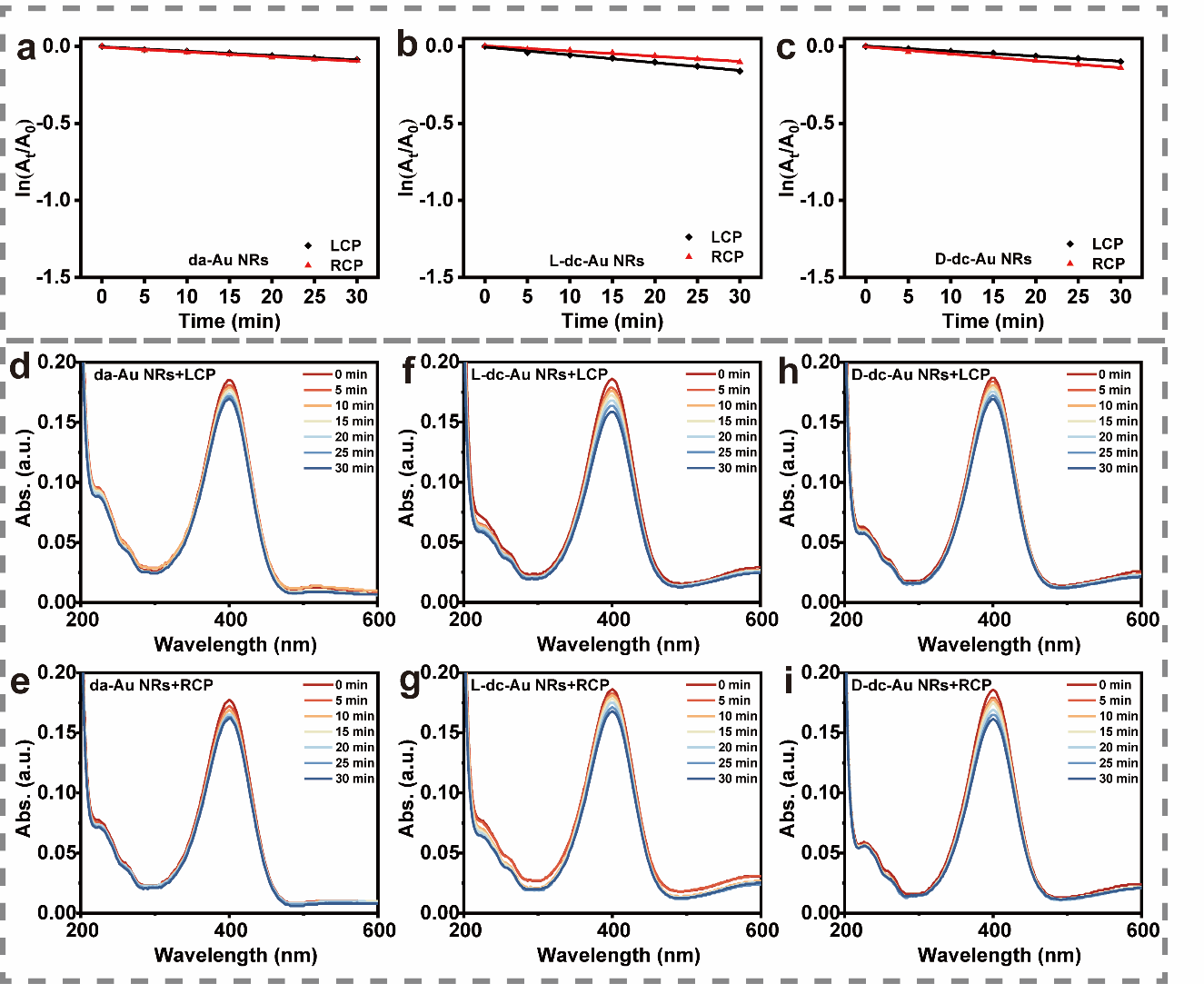


**Figure S16.** (a, b, c) Plots of ln(A_t_/A_0_) as a function of time for the reaction catalyzed by da-Au NRs, L-dc-Au NRs, and D-dc-Au NRs under LCP and RCP illumination, respectively. Time-dependent UV-vis absorption spectra for: (d) da-Au NRs under LCP, (e) da-Au NRs under RCP; (f) L-dc-Au NRs under LCP, (g) L-dc-Au NRs under RCP; (h) D-dc-Au NRs under LCP, (i) D-dc-Au NRs under RCP.


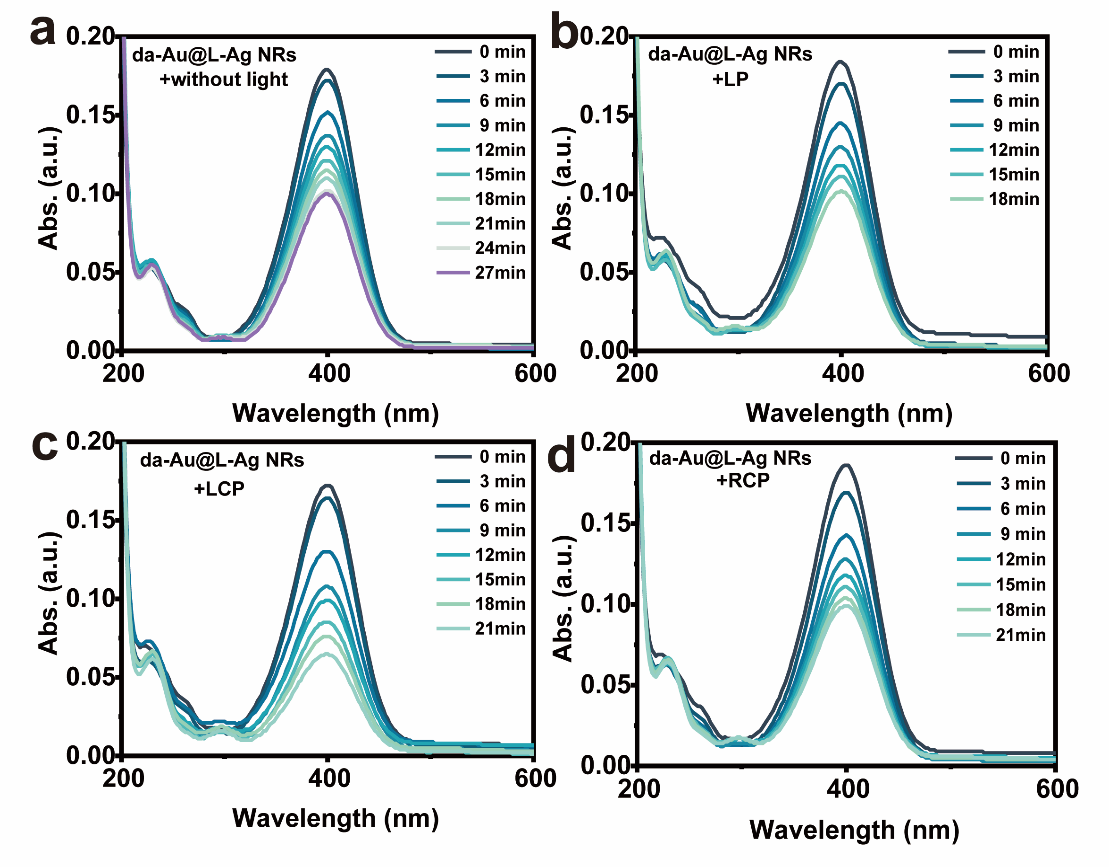


**Figure S17.** Time-dependent UV-vis absorption spectra of the catalytic reaction in the presence of da-Au@L-Ag NRs under dark conditions (a), LP (b), LCP (c) and RCP (d).


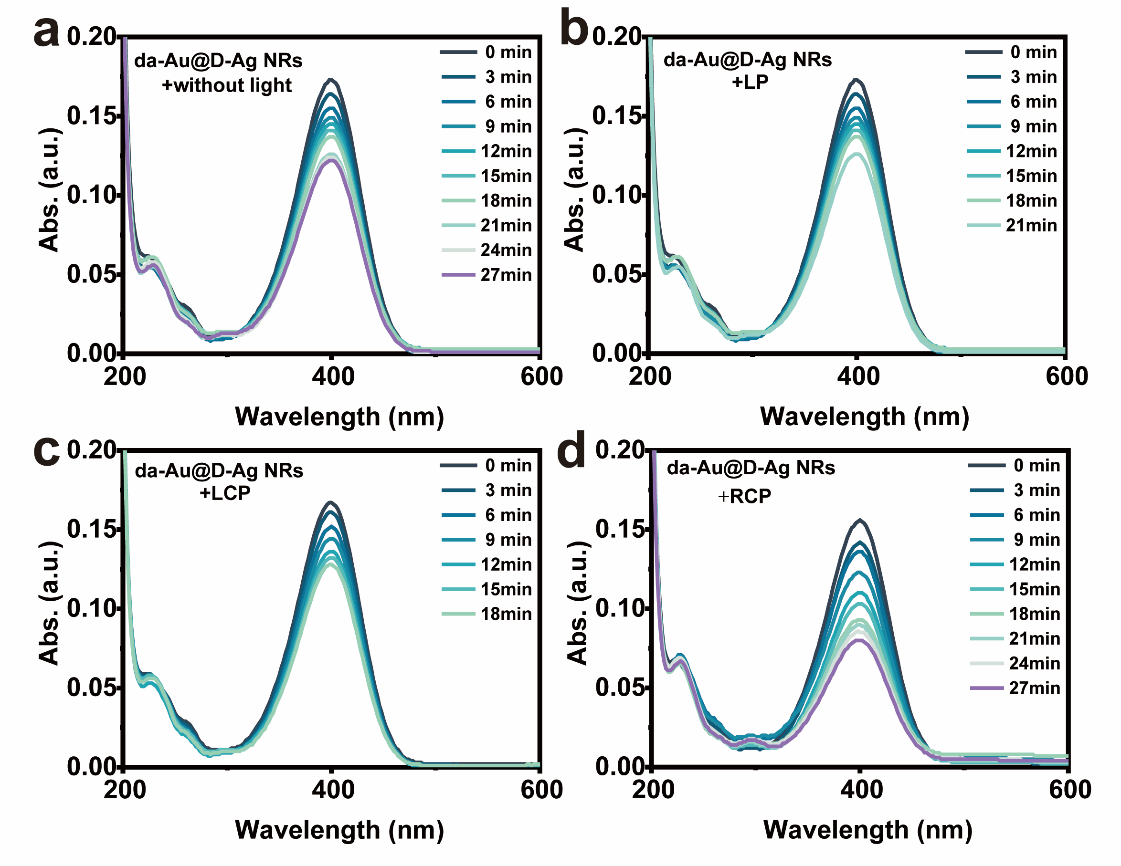


**Figure S18.** Time-dependent UV-vis absorption spectra of the catalytic reaction in the presence of da-Au@D-Ag NRs under dark conditions (a), LP (b), LCP (c) and RCP (d).


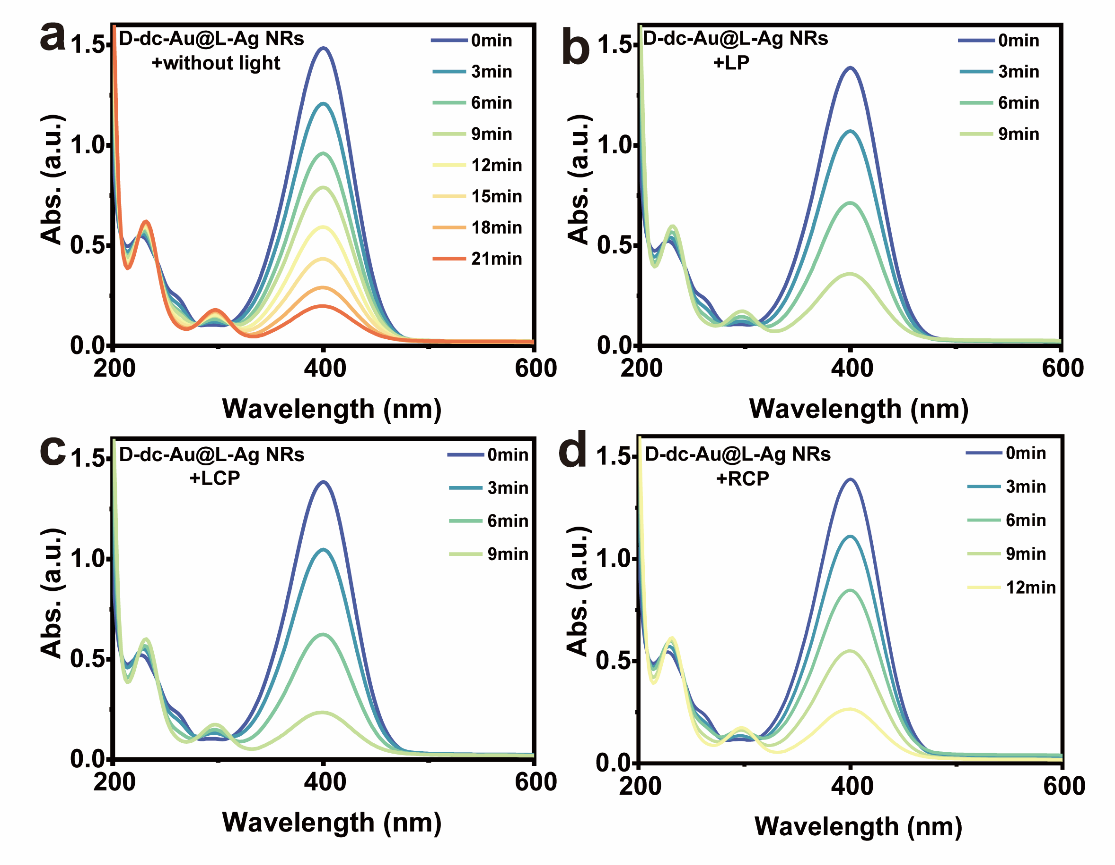


**Figure S19.** Time-dependent UV-vis absorption spectra of the catalytic reaction in the presence of D-dc-Au@L-Ag NRs under dark conditions (a), LP (b), LCP (c) and RCP (d).


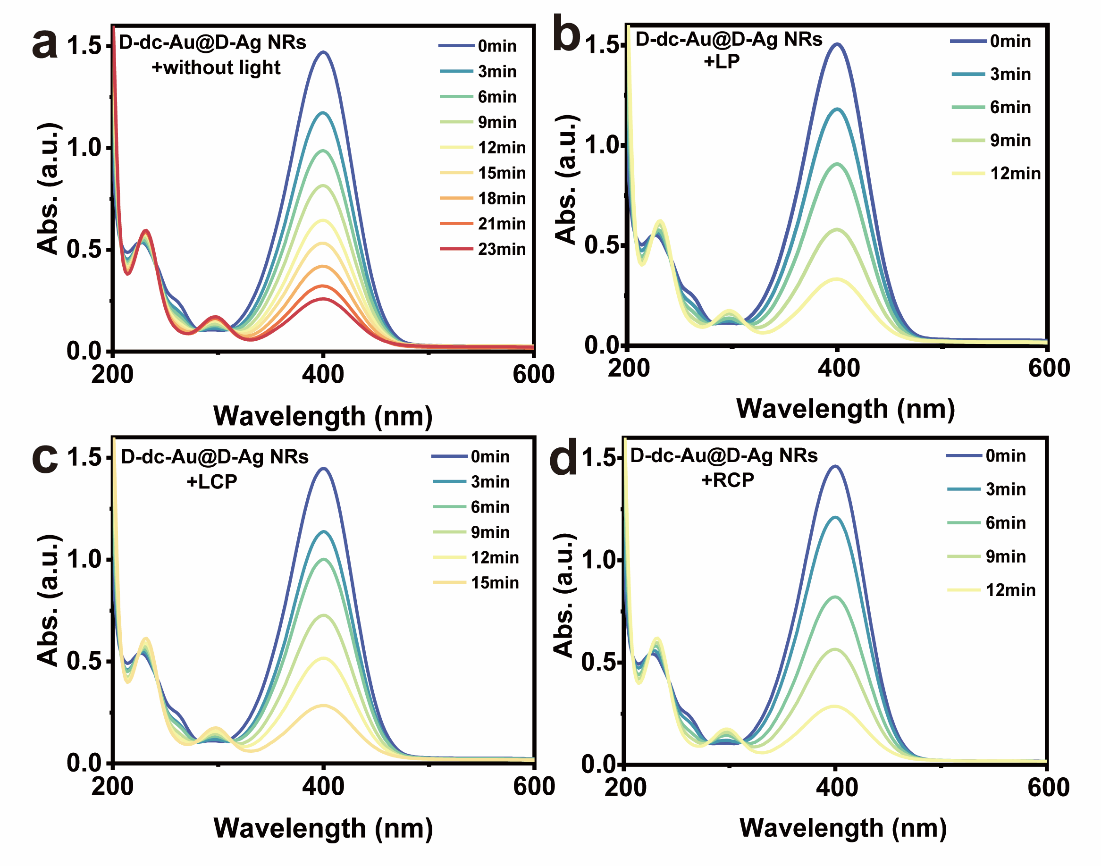


**Figure S20.** Time-dependent UV-vis absorption spectra of the catalytic reaction in the presence of D-dc-Au@D-Ag NRs under dark conditions (a), LP (b), LCP (c) and RCP (d).


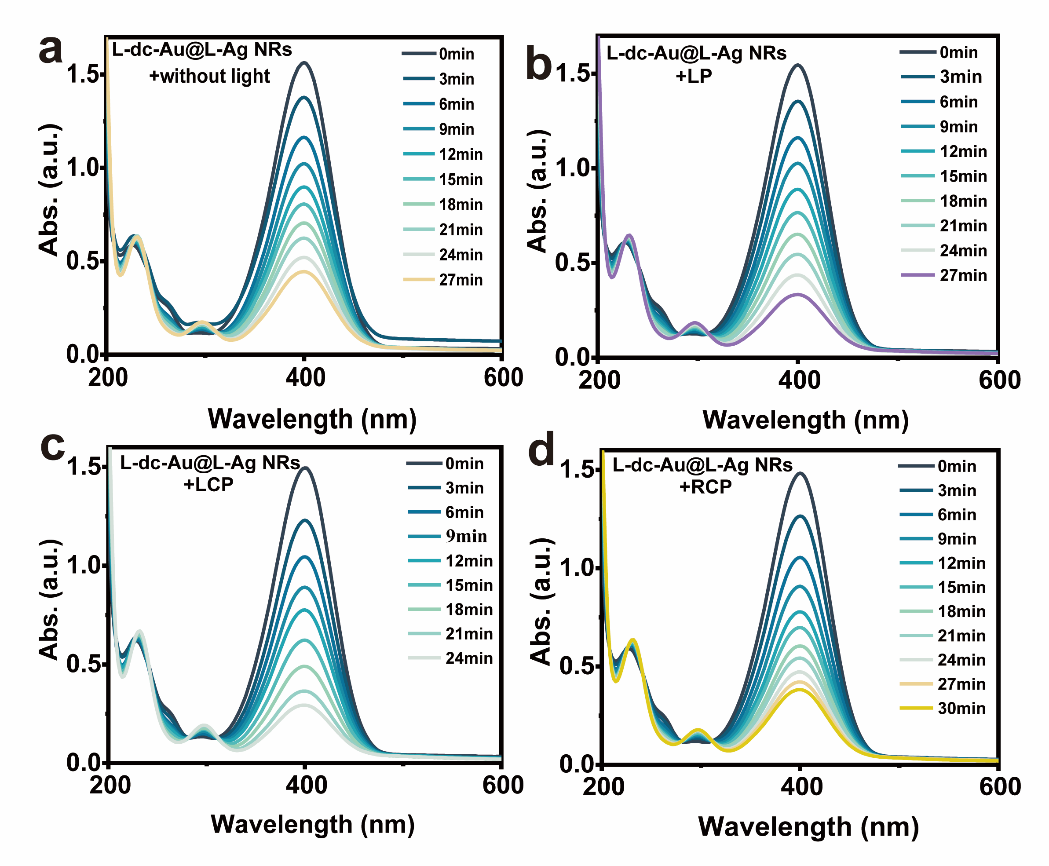


**Figure S21.** Time-dependent UV-vis absorption spectra of the catalytic reaction in the presence of L-dc-Au@L-Ag NRs under dark conditions (a), LP (b), LCP (c) and RCP (d).


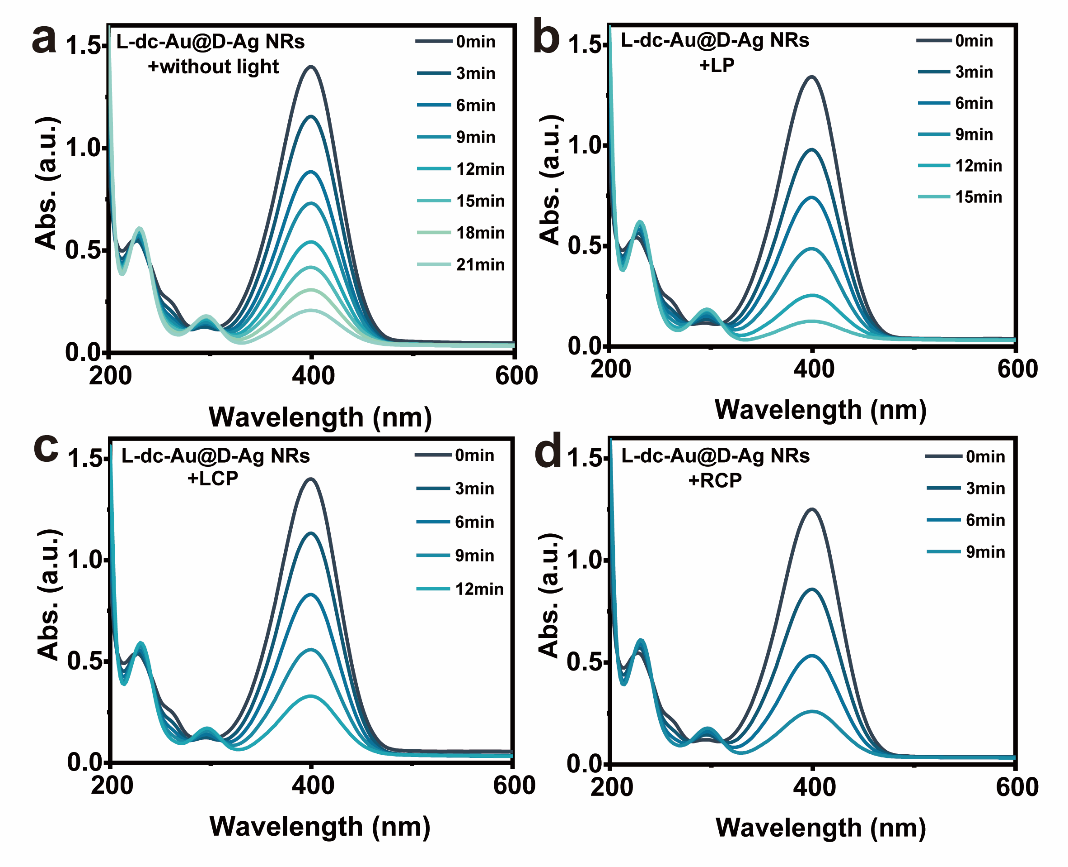


**Figure S22.** Time-dependent UV-vis absorption spectra of the catalytic reaction in the presence of L-dc-Au@D-Ag NRs under dark conditions (a), LP (b), LCP (c) and RCP (d).


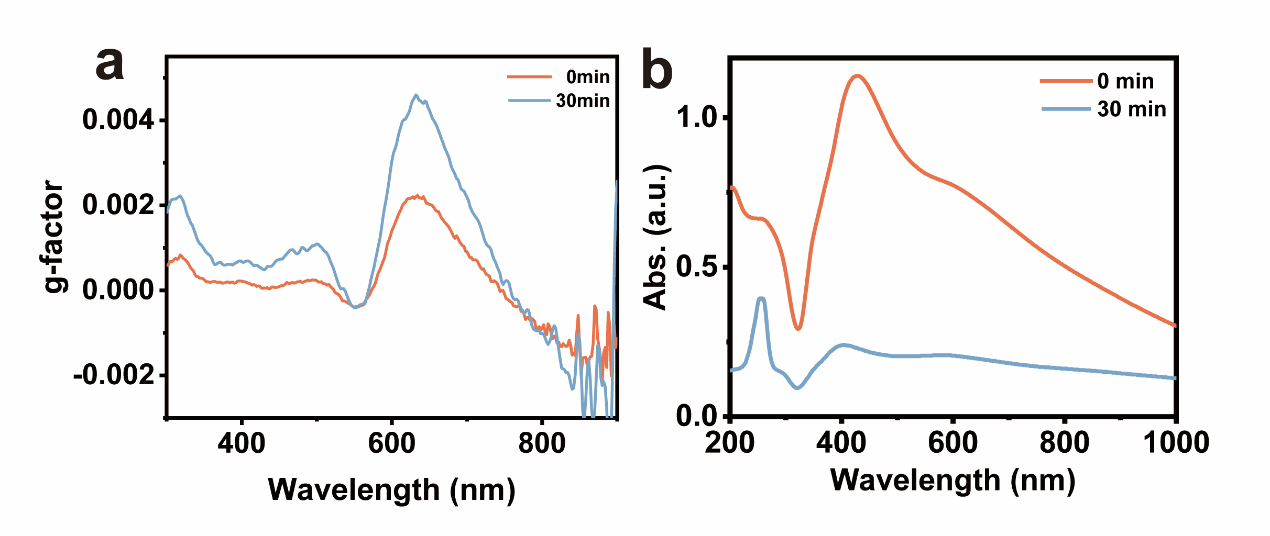


**Figure S23.** (a) g-factor and (b) UV-vis-NIR absorption spectra of D-dc-Au@D-Ag NRs before (0 min, 0 recycle) and after (30 min, 1 recycle) of photocatalysis.

Supplementary Note S1: Definition of scattering, absorption and extinction cross sections:

The optical cross‑sections and the g‑factor were calculated from the simulated electromagnetic fields. The scattering cross section is described as equation S1:

$$\begin{aligned} \sigma_{sc}=\frac{1}{I_{0}}\iint\left( \boldsymbol{n}\cdot\boldsymbol{S}_{\boldsymbol{sc}} \right)dS\#\left( S1 \right) \end{aligned}$$

where $\boldsymbol{n}$ is the normal vector pointing outward from the inside of the structure, $\boldsymbol{S}_{\boldsymbol{sc}}$ is the scattering intensity (Poynting) vector, and $I_{0}$ is the intensity of the incident field.

While the absorption cross section is following equation S2:

$$\begin{aligned} \sigma_{abs}=\frac{1}{I_{0}}\iiint QdV\#\left( S2 \right) \end{aligned}$$

where $Q$ is the power loss density.

The extinction cross section $\sigma_{ext}$ is the sum of $\sigma_{sc}$ and $\sigma_{abs}$. By illuminating the whole system with LCP and RCP light, we can calculate the corresponding $\sigma_{ext}$, ${\sigma_{ext}}^{L}$ and ${\sigma_{ext}}^{R}$, and then extract the g-factor, also called the dissymmetry factor or anisotropy factor. The g-factor (equation S3) is a dimensionless quantity that describes the relative difference in extinction of left- and right-circularly polarized light

$$\begin{aligned} {\sigma_{ext}}^{L}-{\sigma_{ext}}^{R}\#\left( S3 \right) \end{aligned}$$

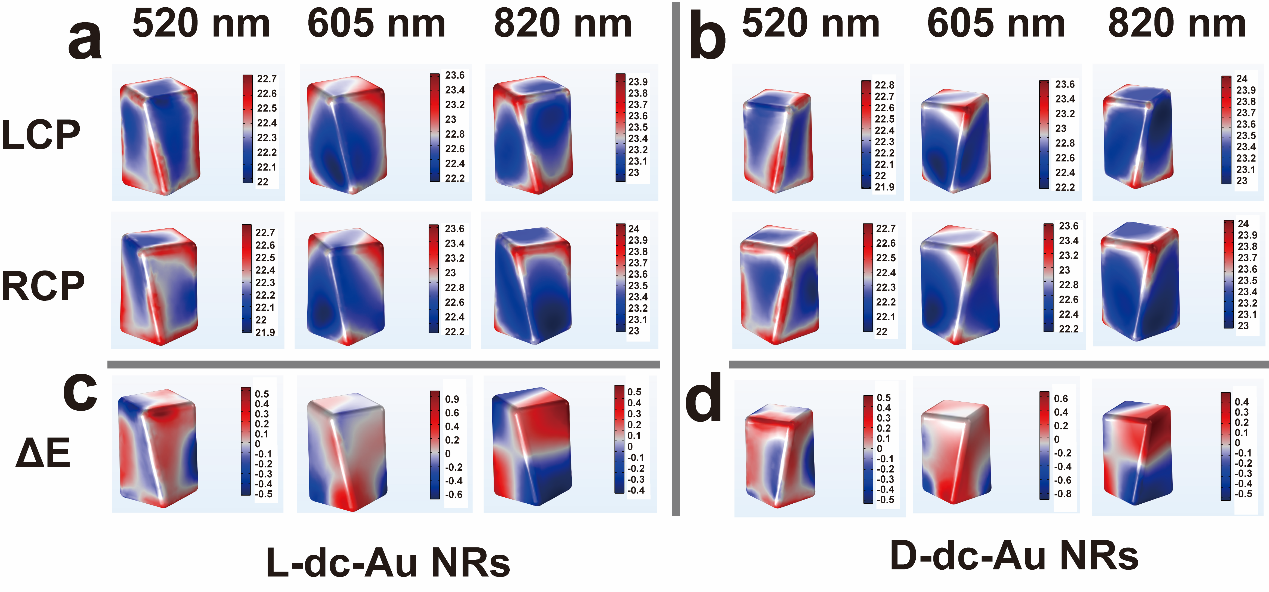


**Figure S24.** Electric field intensities of L-dc-Au (a) and D-dc-Au (b) NRs under LCP and RCP at 520, 605, and 820 nm; (c) and (d) are the corresponding LCP - RCP differences at the same wavelengths for L-dc-Au and D-dc-Au NRs, respectively.


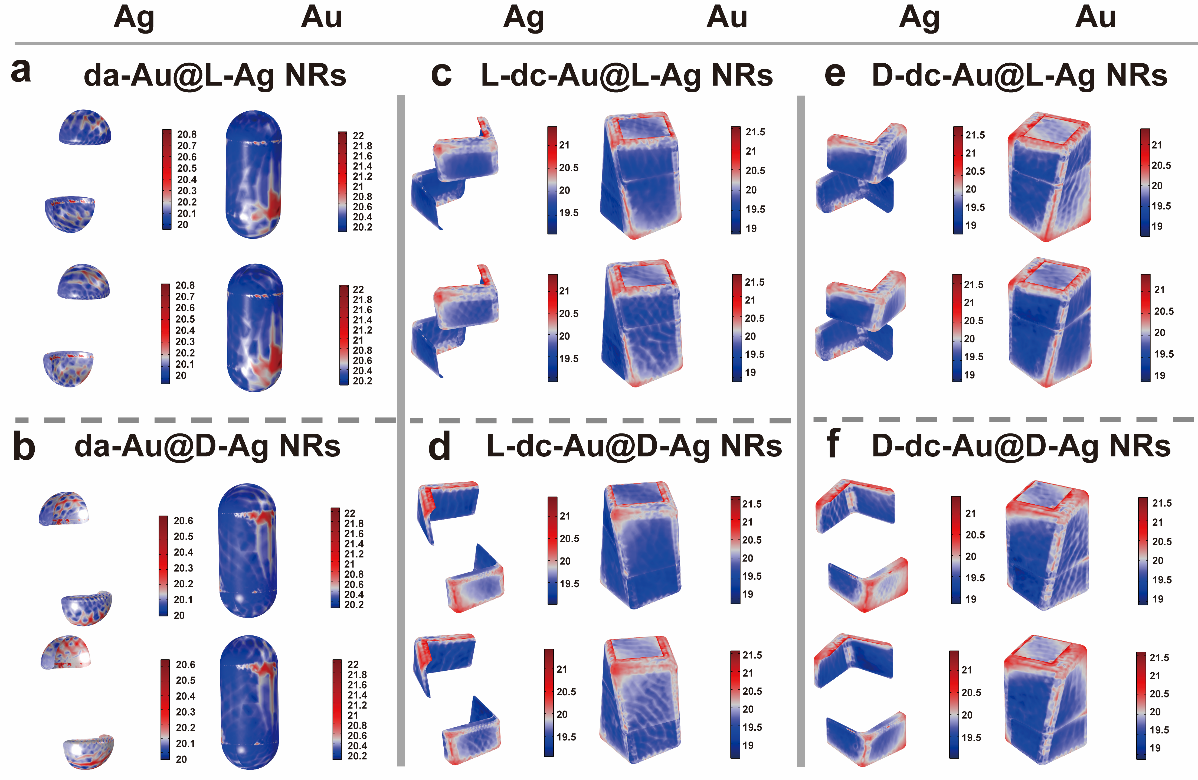


**Figure S25.** The differences in hot electron generation rates (HE Rate) under LCP and RCP for chiral nanoparticles: da-Au@L-Ag NRs (a), da-Au@D-Ag NRs (b), L-dc-Au@L-Ag NRs (c), L-dc-Au@D-Ag NRs (d), D-dc-Au@L-Ag NRs (e), and D-dc-Au@D-Ag NRs (f).


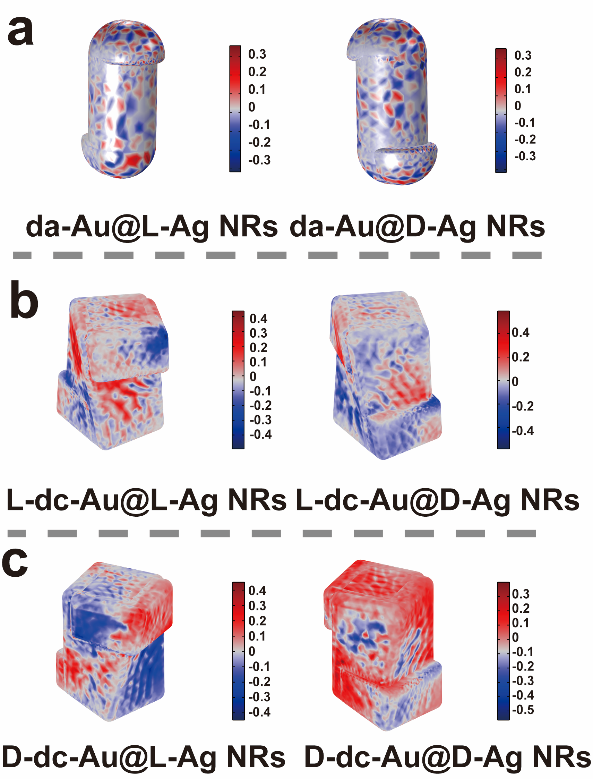


**Figure S26.** The differences in electric field intensity under LCP and RCP for chiral nanoparticles: da-Au@L-Ag NRs (a), da-Au@D-Ag NRs (a), L-dc-Au@L-Ag NRs (b), L-dc-Au@D-Ag NRs (b), D-dc-Au@L-Ag NRs (c), and D-dc-Au@D-Ag NRs (c).


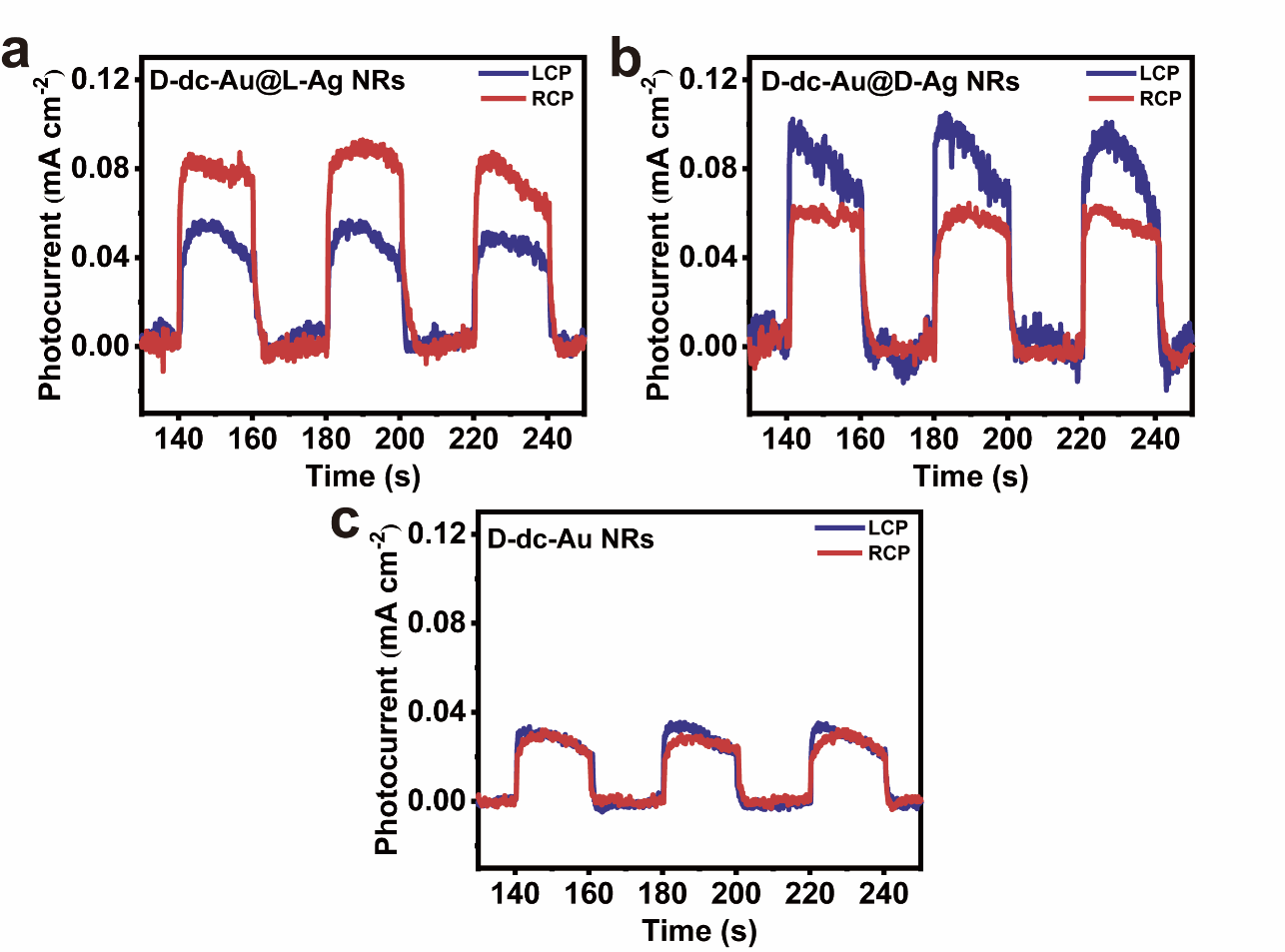


**Figure S27.** Circularly polarized photocurrent responses of (a) D-dc-Au@L-Ag NRs, (b) D-dc-Au@D-Ag NRs and (c) D-dc-Au NRs under LCP and RCP illumination.
